# Chemical Bonding in Monosubstituted Aromatic Molecules from Full-Valence Modern Ab Initio Valence Bond Calculations

**DOI:** 10.1021/acs.jpca.5c06234

**Published:** 2025-11-14

**Authors:** André G. H. Barbosa, João G. S. Monteiro

**Affiliations:** Instituto de Química, 28110 Universidade Federal Fluminense, Niterói 20141-020, Rio de Janeiro, Brazil

## Abstract

Modern ab initio valence bond theory is used to understand the bonding and electronic structure of nine monosubstituted aromatic molecules: fluorobenzene, aniline, toluene, phenol, benzonitrile, benzaldehyde, styrene, nitrobenzene, and benzoic acid. All sigma bonds and in-plane valence lone pairs are treated at the Generalized-Valence-Bond-Perfect-Pairing level while all the pi electrons are described at the Spin-Coupled level including up to all possible spin couplings. Through progressive restrictions on the spin function space, it is shown that in all the molecules considered there is no “pi bonding” between the substituents and benzene ring pi electrons. There are, however, quasi-classical electronic effects that indeed influence the aromatic electron sextet and the sigma bonds in different ways depending on the substituent. These effects are inferred not only by comparing the intrinsic energies associated with the aromatic electron sextet within the group function approach but also by considering the relative position of the centroids of the sigma and pi nonorthogonal singly occupied optimized orbitals. Thus, a comprehensive picture of the electronic effects of the substituents in the benzene ring is presented, identifying and separating the influences associated with the pi and sigma electrons. The obtained model is then compared against experimental physical and reactivity data, giving sound insights into the origin of the electronic effects of substituents on the aromatic ring. Therefore, within a modern ab initio valence bond context, the idea of resonance as a root cause or natural language to describe electronic effects on aromatic molecules should be revised.

## Graphical abstract

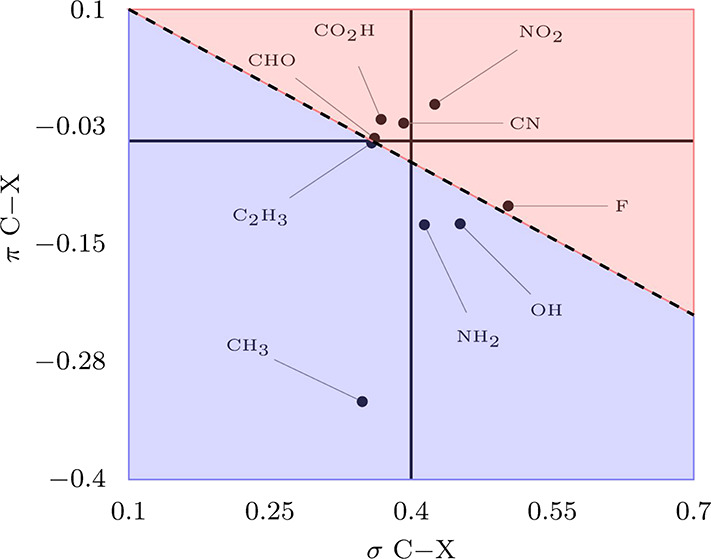

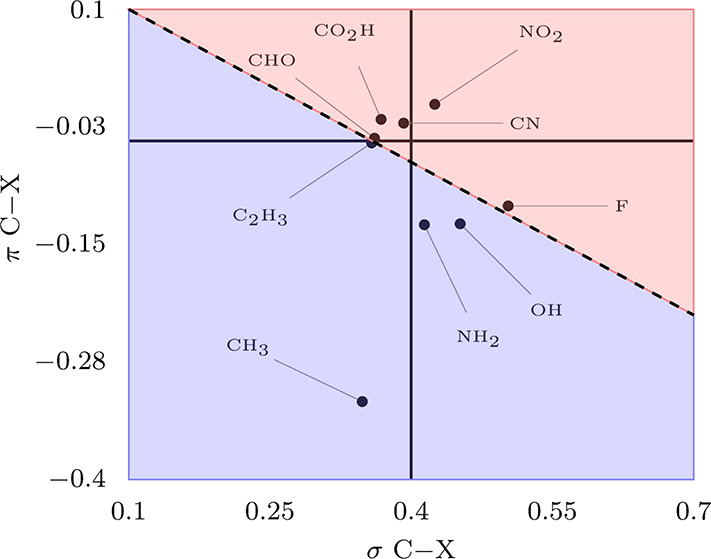

## 1 Introduction

For the last 100 years, chemists have understood the properties of aromatic molecules under the nexus provided by the Lewis theory coupled with the idea of mesomerism, where a chemical structure, i.e., a set of localized bonds and lone pairs, may be understood as a superposition of different bonding patterns. Through this process, the polarity of molecules, the ionic and covalent characters of bonds, and alternative bonding schemes can be qualitatively represented by a superposition of proper chemical structures. Thus, the choice of a particular set of chemical structures for a given molecule is guided not only by the empirical rules of valency and chemical behavior but also by conformity with known experimental data on the associated molecular geometry, polarity, and thermodynamic properties. The qualitative theoretical process beginning with Lewis,^1^ passing through Langmuir,^2^ and concluding with Ingold^3^ and Sidgwick^4^ brought a remarkable order on a jungle of chemical data then available to chemists. Nonetheless, with the advent of quantum mechanics, the Lewis theory of chemical bonding was in a sense upgraded to the valence bond (VB) theory, while the mesomerism concept was upgraded to the resonance concept.^5^ One obvious fact that is often overlooked by chemists is that the VB theory is also a quantitative model, in opposition to the intrinsic qualitative character of the Lewis theory.^6,7^ The fact that the VB theory inherits both the classical chemical structure idea of chemistry together with physical principles from quantum mechanics, with the procedural prevalence of the latter, means necessarily that sometimes its results will not conform to the chemist’s simplistic qualitative ideas. This does not mean that there is something wrong with the VB theory. Apart from the fact that, as any theory, it can be misused, many of its alleged “failures” are not real, as can be verified in the proper literature.^8^ Molecular Orbital (MO) theory appeared roughly at the same time, being concerned with the UV spectra of molecules. Specifically, the MO theory tried to adapt decades of experience of atomic spectroscopy analysis to molecules, developing a language to express its ideas in quantum mechanical terms. Before the rise of computational chemistry in the 1950s, two facts were clear to chemists. The VB theory was uniquely suited to express the concept of chemical bonding and structure in the quantum mechanical language while the MO theory was uniquely suited to interpret UV spectra of molecules in the quantum mechanical language.^9,10^ With the emergence of computational quantum chemistry,^11^ it was realized that a computer program to numerically approximate a value for a given property was much more easily coded within a MO framework than within a VB one. This situation motivated not only an intense work of computational code development of MO theory by many groups (which was very successful) but also a collective effort to conciliate the MO theory with the classical chemical structure and bonding theory (which was not very successful). The powerful and easy-to-use quantum chemical software available today are a consequence of this seven-decade long struggle. Similarly, from the late 1960s to the present time, the VB theory also experienced many theoretical and computational developments, albeit involving a significantly smaller number of research groups. Nonetheless, not only is the VB theory still more computationally demanding than MO for a given numerical accuracy level, but it also does not have a comparable level of automation of its calculational procedures. That is, it may be difficult to use by researchers without some level of specialized knowledge of VB theory and its computational implementation. Nevertheless, the fact that the VB theory is the direct translation of chemical structural theory to quantum mechanics is beyond dispute,^12,13^ holding back nothing of its necessary quantum mechanical foundations, when properly employed.

In this paper, a version of modern ab initio valence bond methodology is employed to understand the electronic structure and chemical bonding of nine monosubstituted aromatic molecules: fluorobenzene, aniline, toluene, phenol, benzonitrile, benzaldehyde, styrene, nitrobenzene, and benzoic acid. They are depicted below in Scheme 1.

**Scheme 1:**
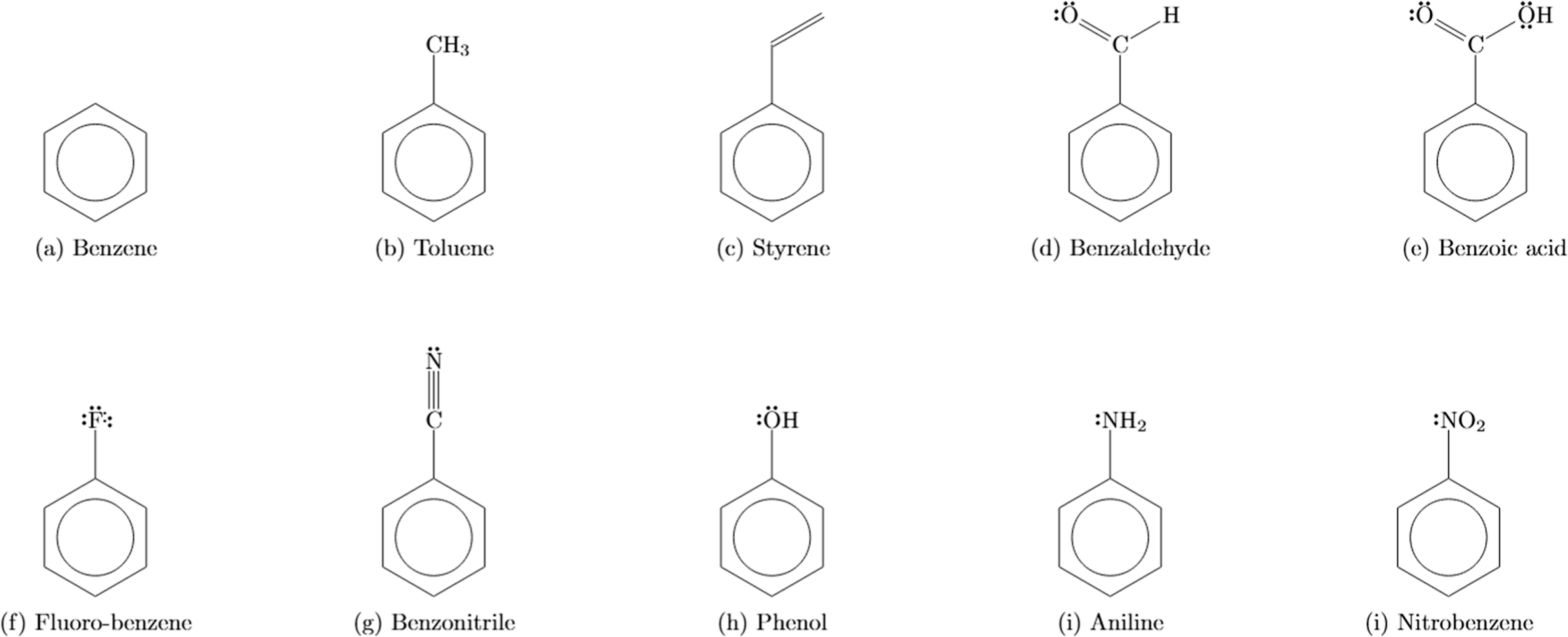
Benzene and the Nine Monosubstituted Aromatic Studied in This Paper

One of the distinctive features of the present work is the inclusion of all sigma and pi bonds in the “active” space; all sigma bonds and sigma valence lone pairs are described as Generalized Valence Bond (GVB) pairs, while pi bonds are described through the Spin-Coupled (SC) approach employing up to the full set of spin functions for a given number of pi electrons. The authors could find two calculations of this kind in the literature, but only for the benzene molecule.^14,15^ In order to take advantage of a full-VB calculation for these molecules, the interpretation must be fine-tuned to detect trends in a relevant set of similar systems, and we tried to do so. We have made attempts to reconcile, as much as possible, the qualitative ideas used in elementary chemistry courses to account for these molecules’ properties with the findings of our calculations. However, given the clear ontological prevalence of quantum mechanics over Lewis’s theory, in light of the results herein presented, we believe that some features of the qualitative description of the electronic structure and bonding of these molecules should be revised.

## 2 Resonance and Delocalization in Aromatic Systems

Lewis theory, particularly under the guise of Valence Shell Electron Pair Repulsion model, assigns electron pairs to specific regions of the molecule, characterizing chemical bonds or lone pairs.^16^ In the situations in which it is necessary to consider alternative chemical bonding schemes, some or all of the electron pairs are assigned to different regions of the molecule. In this case, the electronic structure of the molecule is given by a superposition of mesomeric or resonance structures. Thus, within the context of Lewis theory, the idea of resonance is inextricably associated with the idea of electronic delocalization.^17^

With the advent of quantum mechanics and the ensuing mathematization of the description of the electronic structure, the challenge to apply the new theory to chemistry was set. One of the stark features of many-electron systems, from a quantum mechanical point of view, is that the electrons are indistinguishable.^18^ That is, one cannot label individual electrons, and this fact implies definite consequences for the analytical form of many-electron wave functions. Specifically, acceptable many-electron wave functions for “*N*” electrons must correspond to an antisymmetric representation for the Symmetric Group (*S*_*N*_) under permutations of the “*N*” electron space-spin coordinates.^19^ There are two general ways to accomplish this goal. One can apply the proper projection operators separately for the spatial and spin coordinates; this is the path usually followed by VB-like approaches. Alternatively, one can merge the spatial and spin coordinates into the “spin–orbital” entity and express the wave function in a determinantal form; this is the path usually followed by MO-like approaches. Both approaches generate wave function expansions in which each electron occupies each orbital, one at a time, according to the Pauli Principle. Therefore, in a quantum mechanical antisymmetrized expansion, regardless of the localization or delocalization of the orbitals, electrons are always delocalized. The semantic and physical confusion in the literature arises when “orbital delocalization” is used as a proxy for “electronic delocalization”. The delocalization of orbitals is primarily associated with the combination of two restrictions. First, orbital orthogonality restrictions, which force the extension of functions to all available space, much in the same way as it happens with Laguerre orthogonal polynomial expansions for the hydrogen atoms or with Hermite orthogonal polynomials for vibrational function expansions.^20^ Second, closely associated with the first one, is the standardization of the diagonal form of the kinetic energy operator in the position representation,^21^ as is done for the canonical Hartree–Fock orbitals or for normal mode vibrational function expansions.^22^ An additional contribution to this semantical and physical confusion is associated with the conceptual usage of limited and incomplete atomic basis set expansions.^23^ Physical or chemical significance is often attributed to limited atomic basis sets as representatives of a given atom. Moreover, the energy lowering associated with the existence of nonzero components over basis functions in other atomic centers for orbitals is usually overinterpreted, often leading to the proposal of unsound interpretations of approximate wave functions.^24^ Practical basis sets are always limited and incomplete; we consider that the best way out of this problem, from a conceptual point of view, is to use as interpretation units of what the electrons are doing in molecules functions variationally optimized for singly occupied orbitals, which in a broad valence bond sense are often approximately localized on a given atom allowing a clear physical and chemical interpretation.^25^ Thus, it is known that in the limit of complete basis sets, optimized singly occupied VB orbitals tend to employ basis sets centered in just one atom.^26^ It is not like all the musings of this paragraph are not very well documented in the literature; they are, but are frequently overlooked or misunderstood in many papers and textbooks.^27^ With these brief considerations permeating the background of the interpretation of the results of the present paper, in the next paragraphs, some of the relevant elements of chemical bonding and electronic structure of aromatic molecules will be briefly discussed.

As is well known, the case for the chemical structure of the benzene molecule has been occupying chemists since the 19th century. Accounts for this story up to the ab initio era of quantum chemistry can be found in the literature.^28,29^ Broadly speaking, the VB approach describes the electronic structure associated with the pi electrons as a superposition of the resonance structures. In a practical quantum mechanical sense, “superposition of resonance structures” implies a sort of “configuration interaction”. There are two general meaningful ways in which this superposition of resonance structures is accomplished. The two ways may be divided between a “strict way” and a “non-strict” way.^30^ In the strict way, to each chemical structure corresponds a specifically optimized set of orbitals and spin functions.^12,31–34^ In the nonstrict way, the orbitals are the same in all structures, the chemical bonding patterns being specified by a particular mode of spin coupling.^35,36^ Before the ab initio era, VB calculations were almost always semiempirical, treating resonance in the nonstrict way and without orbital optimization. The benzene molecule was the paradigm for the VB method of resonance. The resonance structures for the benzene molecule were classified as “covalent” or “ionic”. The ionic structures were the ones in which there is charge transfer between atoms, i.e, atoms with a net positive or negative charge for a neutral molecule; the covalent structures were the ones with no charge transfer. Due to the semiempirical approximations done, an accurate estimation of the relative weight of polar and covalent structures was not possible, which kept the VB approach as essentially qualitative for the benzene molecule for decades.^37^ In the meantime, the qualitative MO description for the pi system of the benzene molecule turned out to be much simpler, comprising three delocalized doubly occupied orbitals that within the D_6h_ group consist of a nondegenerate a_2u_ orbital and a degenerate pair of e_1g_ orbitals.^38,39^ It is important to remark that “simpler” is not necessarily the same as “more correct”; it means solely that it is easier to calculate. Thus, from a semiempirical point of view, the MO approach allowed from the outset an easier adaptation of the calculation for benzene to related molecules, i.e., substituted aromatic systems, conveying the idea that the pi electrons of the substituents were conjugated with the benzene ring pi electrons.^40^ This idea was reinforced by particular interpretations of thermochemical measurements.^41^ Additionally, some very influential papers of the early 1950s tried to propagate the (false) idea that the MO depiction of the orbital delocalization between pi systems was, for all practical purposes, equivalent to the electronic conjugation depicted by the superposition of Lewis structures in substituted aromatic molecules.^42,43^ Challenges to these views concerning aromatic substituted molecules were difficult due to the lack of numerically accurate valence bond like wave functions for these systems. With the emergence of computational chemistry in the 1950s, the practicality of the MO approximation guaranteed its increase in popularity in relation to the VB approach. This tendency was reinforced with the beginning of the dissemination of ab initio SCF-MO codes from the 1960s on. Due to difficulties in the computational formulation of the VB theory, the first ab initio calculation of this kind for the pi system of the benzene molecule appeared only in the 1970s.^44^ In the following decade, a more satisfactory Spin-Coupled calculation, comprising full π-orbital optimization was published.^45^ Particularly in this last calculation, it was found that the orbital optimization of the singly occupied nonorthogonal pi orbitals allows an accurate description of the benzene pi system (comparable to a CASSCF calculation of six electrons in six orbitals) without the need for ionic structures. The resulting six singly occupied nonorthogonal orbitals are each one localized above a carbon atom resembling slightly distorted atomic “p” orbitals, an aspect also found in every other closed-shell aromatic system,^46,47^ with the exception of five-center six-electron aromatic systems, in which case it would depend on the details of the particular Spin-Coupled approximation employed.^30^ In light of the last paragraph discussion, it should go without saying that the fact that the pi singly occupied nonorthogonal orbitals of the Spin-Coupled calculations are localized does not mean that the electrons themselves are localized.

Monosubstituted benzene molecules consist of an important class of molecules for testing and applying the Lewis theory and the idea of mesomerism. Differently from benzene, they all have nonzero dipole moments, evidencing an asymmetric distribution of charge. Restricting to the set of molecules calculated in the present paper, the sign and magnitude of the dipole moment was loosely associated with the “electron donation” or “electron withdrawing” effect of the substituent.^3^ For instance, the –OH substituent in phenol is considered to donate electrons to the aromatic system; this is pictured in Scheme 2.

Similar depictions are used for aniline, fluorobenzene, toluene, styrene, and other molecules. The opposite effect, “electron withdrawing”, may be pictured in Scheme 3 by the mesomeric structures associated with the benzonitrile molecule.

Similar depictions are used for benzaldehyde, benzoic acid, nitrobenzene, and other molecules. These qualitative representations are still very popular between chemists. Particularly, important features of electrophilic aromatic substitution reactions can be rationalized with the help of mesomeric structure expansions. First of all, there is the issue of “activation” or “deactivation” of the aromatic ring by the substituent. It is observed that under identical reaction conditions there is a spectrum of reactivities of the aromatic molecules as a function of the substituent. Briefly, those aromatic molecules that react faster than benzene are called “activated” and the substituent is called an “activating group”. Among the molecules calculated herein, this classification applies to aniline, phenol, toluene, and styrene. The opposite effect causes the substituted benzene to react more slowly than benzene with the substituent being called a “deactivating group”. Among the molecules calculated herein, this classification applies to fluorobenzene, benzoic acid, benzaldehyde, benzonitrile, and nitrobenzene. The second issue is a little more complicated, being associated with the fact that different substituents induce different regioselectivities; in one case, a second substituent may preferably enter in the ortho- or para-positions; in another case, the second substituent preferably enters in the meta-positions. Fluorobenzene, toluene, phenol, aniline, and styrene guide substitutions to ortho- and para-positions. Benzaldehyde, benzoic acid, benzonitrile, and nitrobenzene guide substitutions to the meta-positions. All these facts are neatly rationalized using the qualitative Lewis theory as can be checked in standard textbooks.^48^ Nevertheless, it would be desirable to approach sounder physical hypotheses for explanations of these data with a quantum mechanical-based model. Many MO-based calculations on these molecules, of wildly varying quality, were done in the last decades. However, as discussed in the next section, the unitary invariance of molecular orbitals, together with its orthogonality restrictions, limit the amount of chemical insights that can be extracted from them. Full-valence ab initio modern valence bond calculations on these molecules are certainly of high interest. Some of the general questions that we attempt to address in the present paper are the following. What new insights into the nature of the chemical bonds of these molecules can be gained with the present calculations? How and to what extent does the modern ab initio valence bond theory allow a similar rationalization of the body of data on these molecules? What is the distance between the well-known qualitative explanations and an actual quantum mechanical based explanation of the data on substituted aromatic molecules?

## 3 Theoretical Methods

The VB calculations for all nine molecules were carried out with the VB2000 code, which takes advantage of the group function approach together with developments on the matrix element evaluation between configurations comprising nonorthogonal singly occupied orbitals.^14,49,50^ The general form of the VB type wave function employed in this work is presented below and explained in the following.

1 $$\Psi_{S,S_{z}}=A\left[\left(\prod_{i=1}^{n}\varphi_{i}\alpha \varphi_{i}\beta \right)\left(\prod_{p=1}^{n_{pairs}}\left(\psi_{2p-1}\psi_{2p}\right)\left(\alpha \beta -\beta \alpha \right)\right)\left(\prod_{\mu =1}^{N}\left(\psi_{\mu }\right)\sum_{k=1}^{f_{S}^{N}}c_{k}\Theta_{S,S_{z},k}^{N}\right)\right]$$

2 $$f_{S}^{N}=\frac{\left(2S+1\right)N!}{\left(\frac{1}{2}N+S+1\right)!\left(\frac{1}{2}N-S\right)!}$$

“ $\Psi_{S,S_{z}}$ ” stands for a many-electron wave function which is an eigenfunction of the “ ${Ŝ}^{2}$ ” (total spin) and “ ${Ŝ}_{z}$ ” (spin component) operators for the whole set of electrons in the molecule. “ A ” is the antisymmetrizer operator specifying a Slater determinant comprising blocks of three types. The first block is formally common for all space-spin configurations, containing “*n*” core doubly occupied spin–orbitals “φ”. In the present work, these doubly occupied cores consist only of the “core orbitals” per se associated with the inner shell electrons. The second block consists of the valence in-plane pi lone pair or bonds and sigma bonding orbitals, all represented at the generalized valence bond perfect pairing (GVB-PP) level,^51^ comprising pairs of singly occupied nonorthogonal orbitals “ψ”. Each GVB-PP pair makes up a different group, corresponding to mutually strong orthogonal two-electron functions.^52^ In some calculations, for the sake of comparison with expansions involving the full set of spin functions for the pi electrons, the out-of-plane pi electron pairs of the substituent were separated from the aromatic sextet and allocated as a GVB-PP pair. The third type of block contains “*N*” singly occupied orbitals “ψ” and “*f*_*S*_^*N*^” spin functions compatible with the single spatial orbital product “ ${\psi_{\mu }|}_{\mu =1}^{N}$ ”, employed in the description of the out-of-plane pi electrons. This one is called the Spin-Coupled block.^35,53^ “*c*_*k*_” is the spin function coefficient; “ $\Theta_{S,S_{z},k}^{N}$ ” stands for the “*k*th” spin function for “*N*” electrons associated with fixed “ ${Ŝ}^{2}$ ” and “ ${Ŝ}_{z}$ ” eigenvalues.

The analytical form in eq 1 is an alternative presentation of the group function expansion type particularized for the calculations presented in this paper. It is important to note that each group is strongly orthogonal to the other group. This fact is essential for the interpretation of wave functions expressed within the group function approach since it allows a correspondence between the physical interpretation and the constituent energy quantities of a given group of electrons. In the group function approach, each block is at first optimized separately; then a global rotation between different blocks is carried out in order to complete an optimization step. The starting orbitals were obtained from the localization of occupied and virtual molecular orbitals,^54^ followed by a transformation into a singly occupied orbital nonorthogonal basis.^55^

The interpretation of the results of the calculations presented here is dependent on three of the relevant invariant quantities and objects resultant from the calculation: the total energy, the energy of a given group, and the shape of the optimized singly occupied orbitals. One of the most important differences between fully variationally optimized modern ab initio VB wave functions and its counterparts built from Slater determinants composed of orthogonal molecular orbitals is their behavior under unitary transformations of the orbitals. The total electronic energy of a MO-based wave function is invariant in front of unitary transformations within the doubly occupied orthogonal orbital space comprising a Slater determinant.^56^ Even in general multideterminant wave functions, unitary transformations in the doubly occupied orbital space may induce the corresponding transformations in the configuration space in order to maintain the total energy invariant.^57^ The point that is frequently overlooked is that these facts concerning MO wave functions imply the lack of a particular physical significance of individual molecular orbitals, compromising attempts to interpret chemical facts from their shape. On the other hand, fully optimized VB functions do not admit an alternative “unitarily related” orbital basis, its singly occupied nonorthogonal orbital basis being truly invariant. Nonetheless, due to the similarity of the molecules calculated in the present paper, most of the resulting nonorthogonal singly occupied orbitals of the same type are accordingly, at least visually, very similar. While this fact is expected and desirable, being associated with the transferability of understanding between similar bonds in different environments; on first sight, it might bring difficulties in spotting the small but significant differences between related molecules. Therefore, in order to allow a clear comparison between the relevant singly occupied orbitals, one has to employ a unique numerical parameter associated with each orbital. In the present work, with the goal of comparing the effects of different substituents on the aromatic ring, the centroids of the singly occupied nonorthogonal orbitals between a given substituent and the carbon to which it is directly linked are analyzed in detail. The use of centroids of localized orbitals for the assignment of oxidation states of several compounds was recently attempted.^58^ However, the authors used localized doubly occupied orthogonal orbitals, which would make their results dependent on the localization method. Since their centroids are associated with an electron pair, not a single one-electron state, this leads to the intrinsic disadvantage of no clear resolution of the left–right correlation within a given bond. As discussed above, the invariance of doubly occupied orthogonal orbitals under unitary transformation poses a challenge to the uniqueness of their centroids. On the other hand, using optimized singly occupied nonorthogonal valence bond orbitals eliminate this problem since the orbitals are uniquely determined.

Another remarkable invariant of fully optimized VB functions, particularly in the group function form, which is seldom analyzed, is the group energy. In the Supporting Information file, a more detailed description of the group energy and its relationship with the total electronic energy is presented. In our work, the energy associated with the aromatic sextet is evaluated for the benzene and the other nine molecules in question in a specific group partition (see the Results and Discussion section) that treats the six aromatic electrons as a separate group. Since for all molecules the 6-electron groups have the same analytical form for their local wave function and therefore their resulting energy, with very small alterations in the geometric disposition of the ring carbon atoms, the responsibility for the energy changes lies primarily in the global effect of the other groups. In the group function approach, the other groups determine the “external potential” felt by the electrons in a given group. The influence of the other groups is manifested essentially by the intergroup Coulomb and exchange terms (see the Supporting Information) that define different electrostatic environments for each group. This “external potential” in turn leads mainly to altered values of the intragroup one-electron terms (electron kinetic energies and electron–nucleus attractions) that are translated into a lower or higher “group energy”.

The electronic energy “*E* ^(*g*)^” of a group “*g*” of a Spin-Coupled calculation of “*N*” electrons in “*N*” orbitals is given by

3 $$E^{\left(g\right)}=\frac{1}{\Delta^{\left(g\right)}}\sum_{k}^{f_{S}^{N}}\sum_{l}^{f_{S}^{N}}c_{k}^{\left(g\right)}c_{l}^{\left(g\right)}H_{kl}^{\left(g\right)}$$

4 $$\Delta^{\left(g\right)}=\sum_{k}^{f_{S}^{N}}\sum_{l}^{f_{S}^{N}}c_{k}^{\left(g\right)}c_{l}^{\left(g\right)}\Delta_{kl}^{\left(g\right)}$$

in the equations above, “*H*_*kl*_^(*g*)^” stands for the Hamiltonian matrix elements between the space-spin configurations defining the group “*g*” and “*c*_*k*_^(*g*)^” for their coefficients. “Δ_*kl*_^(*g*)^” stands for the overlap between each pair of space-spin configurations and “Δ^(*g*)^” is a normalization factor. In a series of molecules containing an equivalent bonding block, i.e., the aromatic electron sextet, their energy can be computed, yielding additional insights into the intrinsic effects of the ring substituents.

Some remarks on the dimensionality of the spin eigenfunction space are necessary. While the maximum dimensionality of the spin space is fixed, the particular form of the set of spin functions that constitute it is unspecified in front of unitary transformations; that is, it can be expressed in an infinite number of unitarily related basis.^59^ Traditionally, some bases are preferred for computational or chemical convenience, and they can all be transformed into each other when using a complete set.^60^ The calculations presented herein were performed in the Rumer or valence bond basis, which played an important role in suggesting a physical interpretation of the wave functions, as will be explained in the Results section. In spite of this fact, we will not systematically analyze the spin eigenfunction coefficients since it will be proven entirely unnecessary for our purposes. However, in the Supporting Information, we present the calculated spin eigenfunctions, its coefficients, and the orbital overlap matrices for the pi electrons of the soundest wave function for each molecule.

Finally, all the geometries employed in the calculation for the nine molecules considered were optimized at the M06–2*X*/6–311++(d,p) level on the GAMESS program.^61^ The same basis set was employed in the VB calculations.

## 4 Results and Discussion

In Tables 1 and 2, the main numerical results of the calculations are listed. Initially, the results of the calculations will be presented and interpreted for all of the molecules considered. Then, the general trends manifested in the whole set of results will be discussed and related to experimental observations on the chemical and physical properties of these molecules.

**Table 1:** Total Energies at the Hartree-Fock Level and for Different Partitions of the Active Space for Five of the Molecules Calculated^a^

	GVB-PP pairs	SC electrons	SEFs	energies (a.u.)	*E* ^(*g*)^(a.u.)
benzene				–230.756125	
	12	6	5	–230.977591	–6.4413
aniline				–285.804873	
	14	8	14	–286.071099	
	14	8	5	–286.071085	
	15	6	5	–286.068875	–6.4359
phenol				–305.639130	
	14	8	14	–305.904142	
	14	8	5	–305.904114	
	15	6	5	–305.901570	–6.4993
styrene				–307.656605	
	16	8	14	–307.955823	
	16	8	5	–307.955302	
	17	6	5	–307.952232	–6.4656
toluene				–269.802337	
	14	8	14	–270.067605	
	14	8	5	–270.067585	
	15	6	5	–270.067189	–6.4347
fluorobenzene				–329.637264	
	14	8	14	–329.899905	
	14	8	5	–329.899884	
	15	6	5	–329.897844	–6.5918

a The partitions are identified by the number of GVB-PP pairs, the number of SC electrons, the number of spin eigenfunctions (SEFs). In the last column, the intrinsic aromatic electron sextet energy “*E* ^(*g*)^” is shown, associated with the due partition.

**Table 2:** Total Energies at the Hartree-Fock Level and for Different Partitions of the Active Space for the Other Four of the Molecules Calculated^a^

	GVB-PP pairs	SC electrons	SEFs	energies (a.u.)	*E* ^(*g*)^(a.u.)
benzaldehyde				–343.515201	
	16	8	14	–343.818978	
	16	8	5	–343.818826	
	17	6	5	–343.815712	–6.5508
benzonitrile				–322.509944	
	15	8	14	–322.800129	
	15	8	5	–322.799733	
	16	6	5	–322.796771	–6.6453
benzoic acid				–418.433078	
	18	10	42	–418.767619	
	18	10	5	–418.767480	
	18	6 and 4	5 and 1	–418.764367	–6.5431
nitrobenzene				–434.281847	
	18	10	42	–434.643304	
	18	10	5	–434.643242	
	18	6 and 4	5 and 1	–434.640724	–6.6950

a The partitions are identified by the number of GVB-PP pairs, the number of SC electrons, the number of spin eigenfunctions (SEFs). In the last column, the intrinsic aromatic electron sextet energy “*E* ^(*g*)^” is shown, associated with the due partition.

In all VB calculations, all valence electrons were included in the active space. All sigma bonds and in-plane pi bonds and lone pairs were described at the GVB-PP level as pairs of singly occupied nonorthogonal orbitals. The out-of-plane pi electrons were described in a variable way depending on the molecule. From the variational point of view, the highest level for the pi electrons consisted of considering all of them together in a Spin-Coupled block with all possible spin eigenfunctions included. The next level still considers all pi electrons in the Spin-Coupled block but restricts the number of spin eigenfunctions to the five necessary to properly describe the aromaticity of the benzene moiety. The next more restrictive level isolates the aromatic electron sextet from the substituent electrons, putting the respective electrons in separate groups. In order for the reader to understand the finer differences between different calculations for the same molecule, one representative example will be explained at full length, reserving additional pertinent brief comments for the other molecules calculated. Hopefully, this procedure will help to clarify the significance of all of the results presented.

### 4.1 Phenol

The geometry for phenol was optimized at the M06–2*X*/6–311++(d,p) level, yielding a planar nuclear configuration, as expected. In this molecule, there are 14 core electrons allocated in seven doubly occupied orthogonal orbitals. The remaining 36 valence electrons are allocated in 18 doubly occupied valence orbitals in a Hartree–Fock calculation whose energy (−305.639130 au) is shown in Table 1. These 36 valence electrons were initially separated in two sets; 28 electrons associated with the sigma bonds and in-plane valence lone pairs and 8 electrons associated with the aromatic electron sextet plus a pi-like lone pair of the hydroxyl moiety. These orbitals and a comparable set of unoccupied orbitals were localized through the Edmiston-Ruedenberg procedure in order to generate a guess for the VB wave function.^56^ There are six C–C sigma bonds, five C–H sigma bonds, one C–O σ bond, one O–H σ bond, and one O in-plane valence lone pair comprising 28 sigma electrons described by 28 different singly occupied nonorthogonal orbitals corresponding to 14 GVB-PP pairs, as indicated in Table 1. Eight pi electrons were allocated in eight singly occupied nonorthogonal orbitals, which in a full Spin-Coupled expansion are associated with 14 spin eigenfunctions. This partition corresponds to the converged total energy of −305.904142 au, comprising an amount of 0.265012 au of “non-dynamic” correlation energy. From Table 1, this partition may be indicated briefly in the case of the phenol molecule as partition (14,8,14). A common feature associated with this higher Spin-Coupled level, for all of the molecules considered, is the larger importance of five spin eigenfunctions in relation to the others. The characteristic feature of these important spin eigenfunctions is that in all of them the substituent pi electrons are coupled in a singlet, apparently standing apart from the aromatic electron sextet. That is, the five spin-coupling modes necessary to describe the ring aromaticity, regardless of the substituent, are always the most important. In other words, the combined Chirgwin-Coulson weights of these five space-spin configurations is,^62^ for all the molecules considered, always larger than 99%. This fact may be interpreted as indicating a lack of covalent bonds (stabilizing the singlet spin-coupling interaction between two adjacent atoms) between the aromatic electron sextet and the pi electrons of the substituent. In order to test this interpretation, two more calculations were performed. The first of them, for the phenol case, consists of maintaining the same orbital partition but excluding the spin eigenfunctions that couple the substituent electrons with those of the aromatic sextet into a singlet; only the five spin eigenfunctions necessary for the description of the aromaticity are retained. This partition is labeled (14,8,5). The resulting energy for this calculation, −305.904114 au, is essentially identical to the calculation comprising 14 spin eigenfunctions; note that, in this case, the singly occupied pi orbitals of the hydroxyl group remain nonorthogonal to the singly occupied aromatic pi orbitals. In the second additional calculation, the pi lone pair of the phenol hydroxyl is completely isolated from the aromatic sextet, being placed in a separate strongly orthogonal group partition (15,6,5). The resulting energy, −305.901570 au, is nearly identical to that of the highest Spin-Coupled level; the difference of less than 0.003 au (1.88 kcal/mol) arises mainly from the strong-orthogonal restriction. This situation is similar to that of the pi system of the 1,3-butadiene molecule; a careful analysis showed that, contrary to common belief, there is no conjugation between the two adjacent pi bonds in the 1,3-butadiene molecule, which is quite the opposite.^63^ Returning to the phenol molecule, apart from the aforementioned small energy difference, another manifestation of the strong-orthogonal restriction between the aromatic sextet and the pi electrons of the substituent may be pictured by comparing the shape of the singly occupied pi orbitals of the substituent. In the case of the phenol molecule, this is shown in Figure 1, and an identical effect occurs for the fluorobenzene molecule. In all orbital pictures, the contour value was fixed in 0.1 using the MacMolplt software.^64^

**Figure 1:**
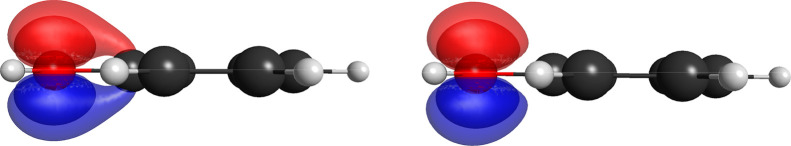
Effect on the strong-orthogonal restriction on the pair of singly occupied nonorthogonal pi orbitals of the hydroxyl moiety for the (14,8,5) (left) and (15,6,5) (right) partitions for the phenol molecule.

From the irrelevant energy difference between the (14,8,14) and (14,8,5) partitions, it can be unmistakably concluded that there is no pi bonding between the hydroxyl group and the aromatic sextet system. Alternatively, this conclusion may be rephrased, saying that resonance structures that describe a pi bonding between oxygen and carbon in this molecule have negligible coefficients. The small stabilization of the (14,8,5) partition in relation to the more restrictive (15,6,5) partition should be attributed to quasi-classical effects associated with orbital delocalization in the absence of a strong-orthogonal restriction,^63^ as depicted in Figure 1.

### 4.2 Aniline and Fluorobenzene

Essentially, everything that was discussed for the phenol molecule above also applies to these two molecules, as can be seen from the results in Table 1. The only noticeable difference is the shape of the singly occupied pi orbitals of aniline, due to the fact that the two hydrogens of the amino moiety are slightly off the benzene molecular plane in the optimized geometry. These are shown in Figure 2. The singly occupied π orbital over the carbon atom adjacent to the nitrogen atom in the aniline molecule is shown in Figure 3. This orbital is very similar to all carbon pi singly occupied orbitals for the other molecules. Similarly, the pair of singly occupied nonorthogonal orbitals for the C–H, which are visually similar for all molecules, is shown for the fluorobenzene molecule in Figure 4.

**Figure 2:**
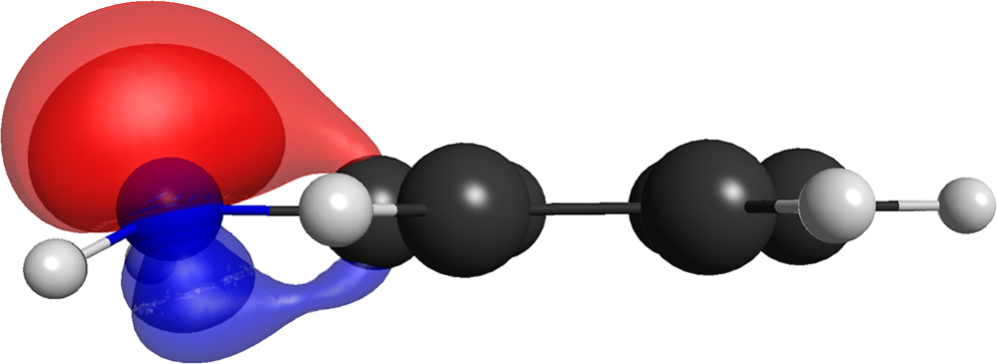
Pair of singly occupied nonorthogonal pi orbitals for the lone pair of the aniline molecule for the (14,8,5) partition.

**Figure 3:**
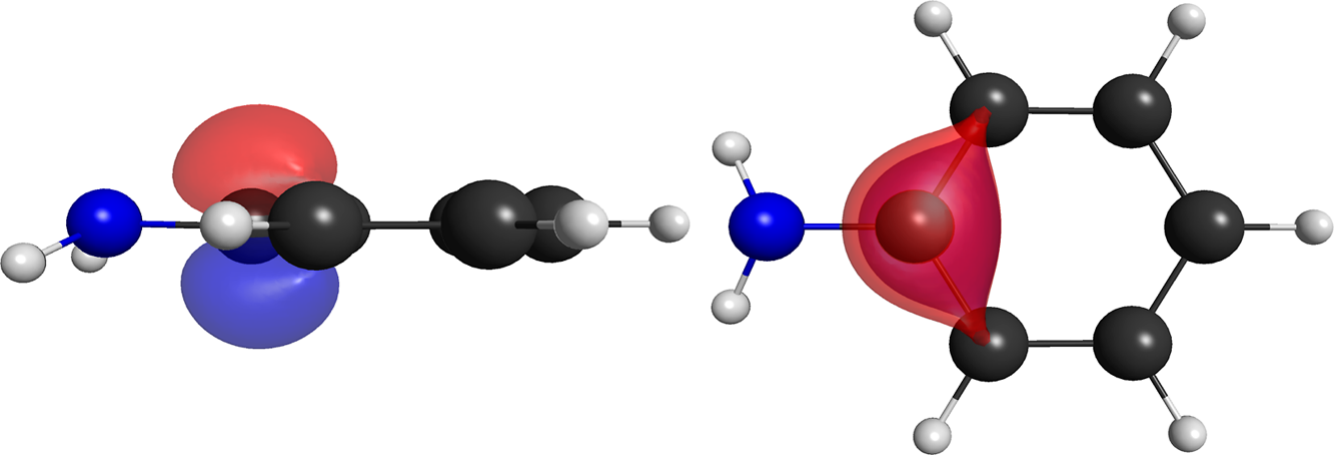
Side and top views of one of the visually equivalent singly occupied nonorthogonal pi orbitals of the aniline molecule for the (14,8,5) partition.

**Figure 4:**
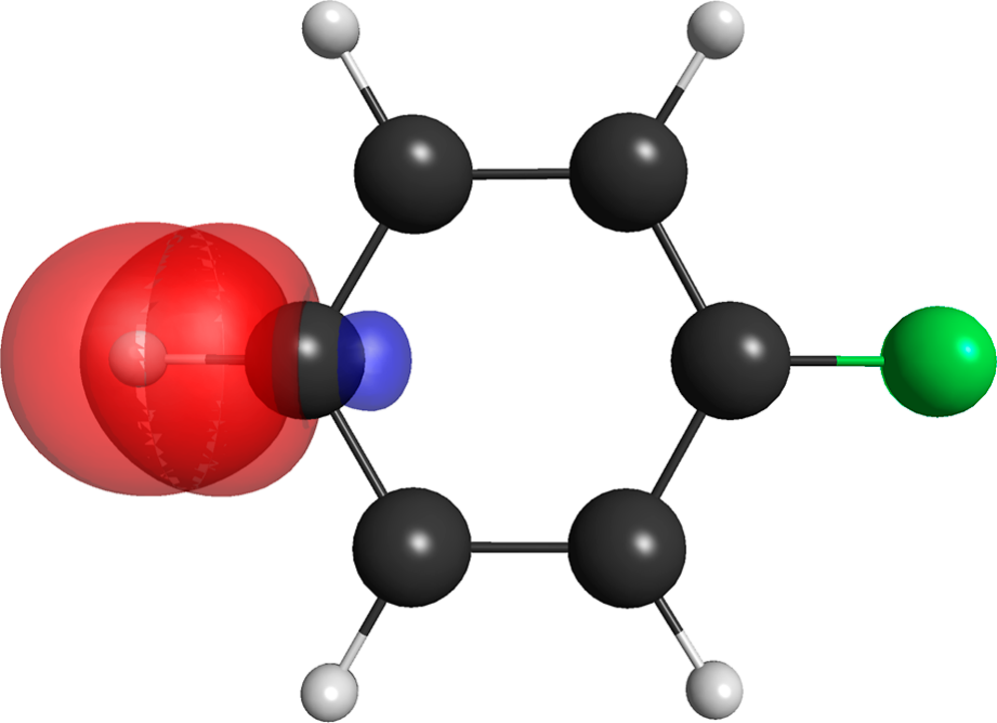
Pair of singly occupied nonorthogonal orbitals for the sigma C–H bond in the fluorobenzene molecule for the (14,8,5) partition.

### 4.3 Styrene

In spite of many experimental and computational studies on the molecular structure of styrene, there is still some uncertainty regarding the planarity of the styrene molecule in the gas phase.^65^ Two local minima were found at the M06–2*X*/6–311G­(d,p)++ level, differing essentially in the torsional angle (31° and 8°) between the benzene ring plane and the vinyl group plane. Nonetheless, the obtained qualitative results concerning the bonding of its pi electrons to the aromatic sextet are still identical to those of phenol, aniline, and fluorobenzene, as can be seen in Table 1 for the M06–2*X*/6–311G­(d,p)++ minimum geometry having an 8° torsional angle between the benzene ring and vinyl group planes. That is, there is no covalent bonding interaction between the pi electrons of the vinyl group and the aromatic sextet. The same qualitative results are obtained for the secondary minimum having a 31° torsional angle.

### 4.4 Toluene

In this case, one has to choose the substituent C–H bond most parallel to the ring pi system orbitals to compose the eight electron SC group partition (14,8,14) in Table 1. The restriction of the number of spin functions to the five necessary to describe the ring aromaticity partition (14,8,5) barely alters the total electronic energy. The additional imposition of strong orthogonality between the C–H σ bond and the aromatic electron sextet partition (15,6,5) led to the smallest energetic cost in comparison to all the molecules calculated herein. Thus, for this molecule, it makes no physical sense whatsoever to put together in the same Spin-Coupled space the aromatic electron sextet and one or more sigma C–H bonds of the methyl group. Previous experience with optimized valence bond wave functions indicates that the C–H sigma bonds near pi bonds become strongly orthogonal upon the variational optimization of the orbitals.^17,24,66^ The only situation where this does not happen is when the molecule is fluxional,^67,68^ which is not the case for the molecules in this paper. Adding, on top of all that, the results concerning the lack of pi bonding between lone electron pairs and aromatic sextet electrons for all the other molecules, one can safely assume a priori that there is no pi bonding between the methyl moiety and the aromatic ring in the toluene molecule. Toluene is, from the present calculations, the strongest “pusher” of electrons in the pi space. This effect is closely related to the strong orthogonal separation between the aromatic electron sextet and the C–H bond of the methyl moiety. From a physical point of view, this effect has already been described with similar wave functions in simpler molecules.^69^ This issue will be dealt with in comparison with the other molecules in a later discussion in this paper regarding the physical interpretation of the substituent effects on the aromatic electron sextet. The singly occupied orbitals involved in C–C sigma bonds are visually very similar in all molecules calculated here; as an illustrative example, a pair for the toluene molecule is shown below in Figure 5.

**Figure 5:**
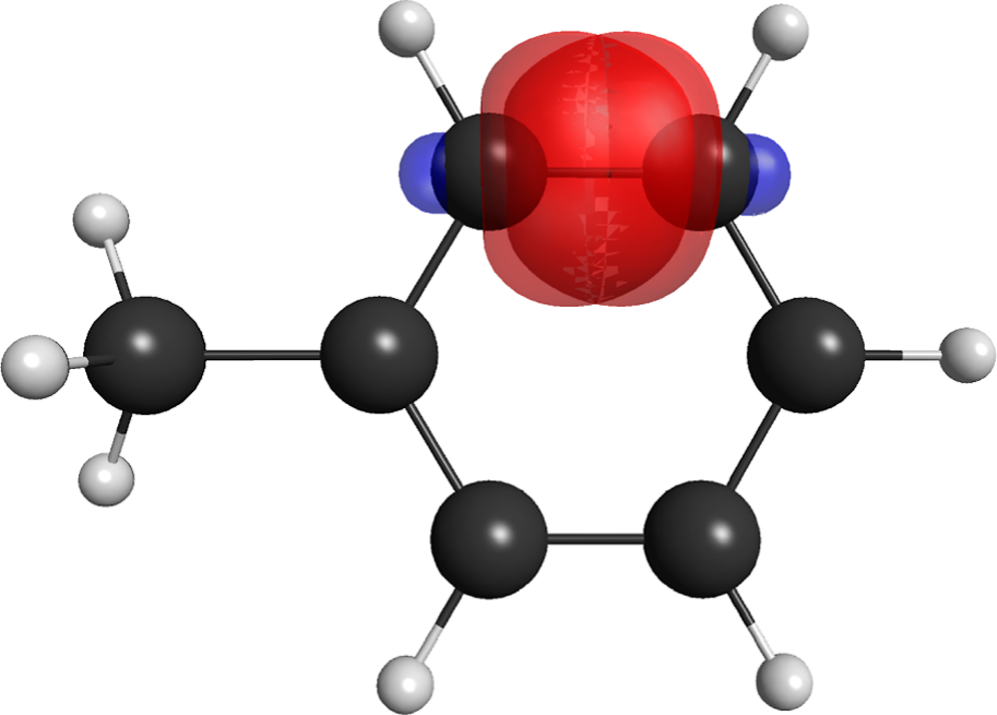
Pair of singly occupied nonorthogonal orbitals for the C–C σ bond of the toluene molecule for the (15,6,5) partition.

### 4.5 Benzaldehyde and Benzonitrile

The results for these molecules are given in Table 2. Contrary to the molecules discussed up to now, all of the systems in Table 2 are considered to “withdraw pi electrons” of the benzene moiety. However, apart from the different partitions, almost everything that was discussed for the above molecules regarding the absence of pi bonding between the substituents and the aromatic electron sextet also applies to these two molecules, as can be seen from the numerical results in Table 2. A hardly noticeable difference is associated with the benzonitrile molecule in the energy difference between partitions (15,8,14) and (15,8,5); it is the largest between the molecules calculated herein, in spite of still being very small (0.25 kcal/mol). A picture for the pair of optimized singly occupied nonorthogonal pi orbitals of the CN bond in the benzonitrile molecule is shown in Figure 6.

**Figure 6:**
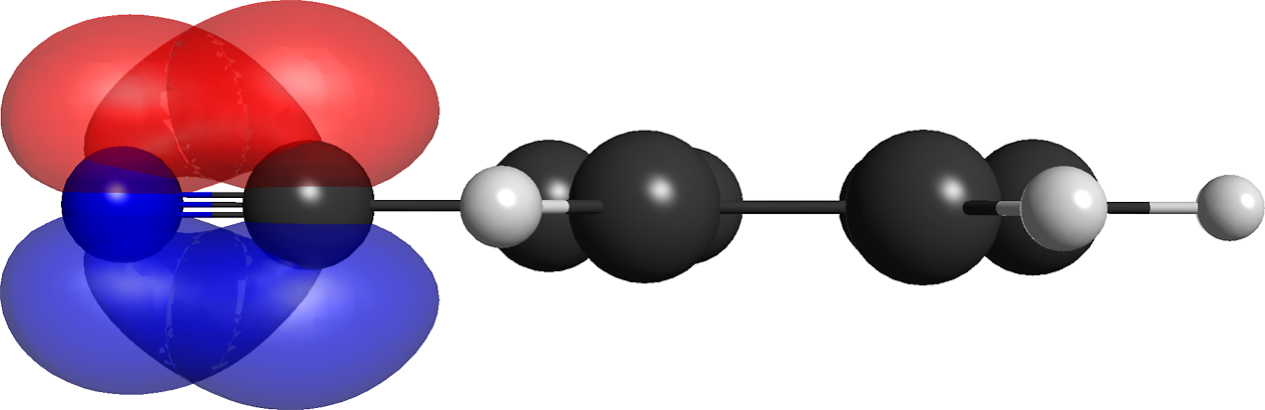
Pair of singly occupied nonorthogonal orbitals for the CN out-of-plane π bond of the benzonitrile molecule for the (15,8,5) partition.

### 4.6 Nitrobenzene

There are two important differences in this case in comparison with the previous molecules (Figure 7). First, there is the presence of four pi electrons in the substituent, a feature shared with the benzoic acid. Second, associated with the first, there is a special pi bonding scheme within the substituent that is exclusive to the nitro group. It can be seen in Table 2 that the highest Spin-Coupled level for the nitrobenzene molecule comprises 10 electrons and 42 spin eigenfunctions partition (18,10,42). The removal of all spin eigenfunctions describing singlet coupling between a pi electron in the substituent and a pi electron in the aromatic sextet partition (18,10,5), barely alters the total energy. As already discussed above, this fact is a definite indication that there is no pi covalent interaction between the nitro moiety and aromatic ring. The strong-orthogonality restriction cost of isolating the pi electrons of the nitro group from the aromatic ring is essentially similar in almost all molecules, including nitrobenzene. The pi chemical bonding within the nitro group is somewhat different than usual, consisting in one of the cases where there is a dissonance between the perfect-pairing and the strong-orthogonal approximation.^55^ Specifically, if one employs the strong-orthogonal approximation, upon orbital optimization, the singly occupied orbitals rearrange to yield a spurious biradical structure for the pi electrons of the nitro group. However, if one keeps the four singly occupied orbitals nonorthogonal to each other in the group, the optimized wave function describes two identical pi bonds associated with only one spin eigenfunction. In Figure 7 below, the singly occupied nonorthogonal of one of the pi bonds in the nitro moiety is shown.

**Figure 7:**
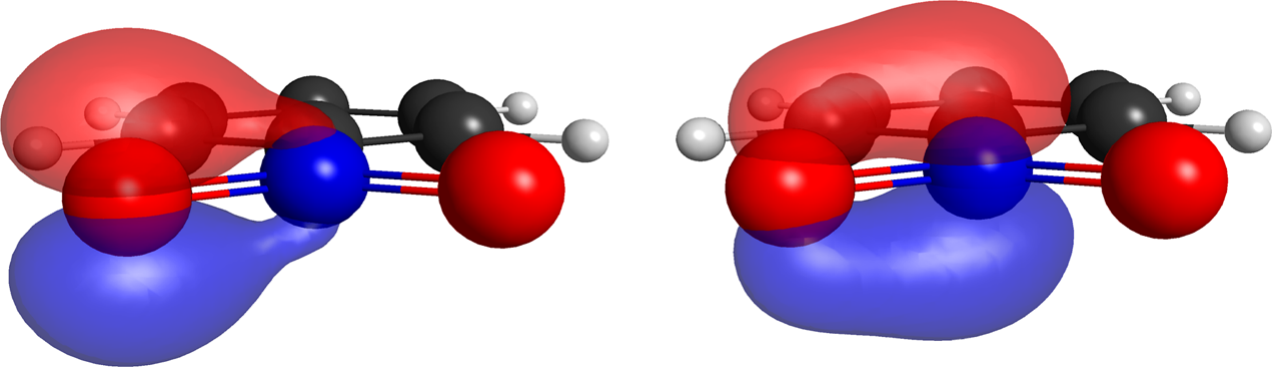
pair of singly occupied nonorthogonal pi orbitals of the nitro moiety in nitrobenzene, comprising one π bond partition (18,10,5). An exactly equivalent pair, nonorthogonal to the one depicted above, is associated with the other oxygen and the central nitrogen atom.

A subsequent paper dealing specifically with nitro aromatic molecules with other additional substituents is in preparation, allowing a more comprehensive discussion of these features and their chemical and physical consequences.

### 4.7 Benzoic Acid

In this case, there are also four pi electrons in the substituent, but the other features regarding the lack of a pi covalent bond between the substituent and the aromatic ring remain, as can be checked from the results in Table 2. The contrast between the CO π bond and the hydroxyl pi lone pair of the carboxyl group of the benzoic acid molecule is shown in Figure 8, below.

**Figure 8:**
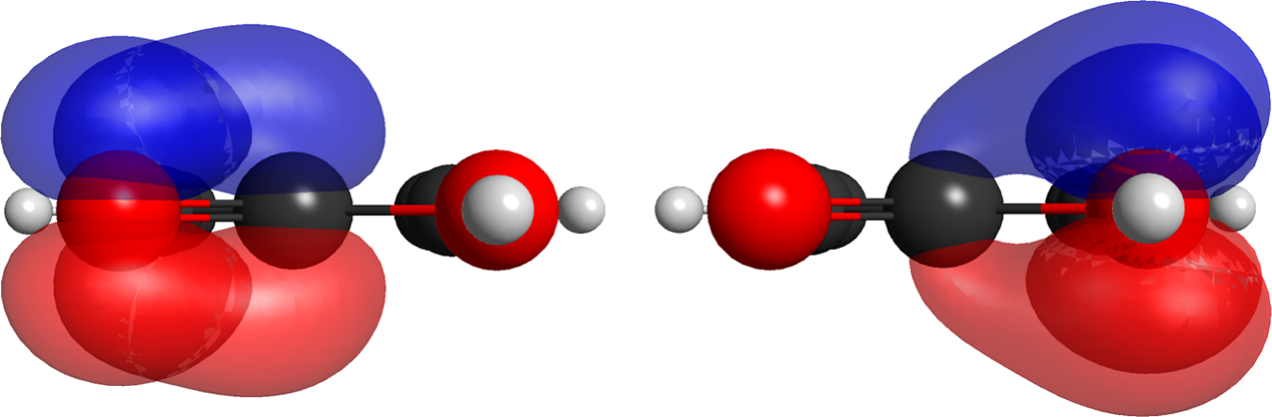
On the left, the pair of optimized singly occupied nonorthogonal pi orbitals of the CO bond in benzoic acid; on the right, the pair of optimized singly occupied nonorthogonal pi lone pair of hydroxyl’s carboxyl of benzoic acid, both referring to partition (18,10,5).

We consider that for all molecules, with the exception of toluene, the more physically and chemically sound wave functions are the ones where the pi orbitals of the substituent are still nonorthogonal to the pi orbitals of the aromatic system but with a reduced set of spin eigenfunctions, as indicated in the second VB entry for each molecule in Tables 1 and 2. In the case of toluene, the wave function that isolates the aromatic electron sextet from the C–H bonds of the substituent partition (15,6,5) is clearly the more reasonable approximation. The lack of actual pi bonding between the substituents pi electrons and the aromatic sextet is a stark manifestation of the particular resiliency of the aromatic electron sextet, which is an experimentally observed and theoretically established fact.^15,70^

## 5 A Physically and Chemically Sound Model for the Substituent Effects

It is important to note that in all calculations all the valence sigma bonds and lone pairs were correlated as GVB-PP pairs. This means that the effective potential to which the pi electrons were submitted is more accurate than the usual Hartree–Fock potential. There are cases where the proper bonding description of the sigma electrons has a significant qualitative and quantitative effect for a proper description of the bonding of the pi electrons.^55,71,72^ Therefore, in the ensuing analysis, features of both the pi and sigma electrons as manifested by the, respectively, optimized singly occupied nonorthogonal orbitals will be considered in relating the results of our calculations with the observations concerning these molecules.

**Scheme 2:**
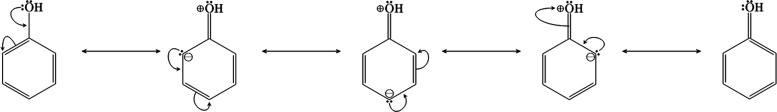
Depiction of the Mesomeric Structures of the Phenol Molecule

**Scheme 3:**
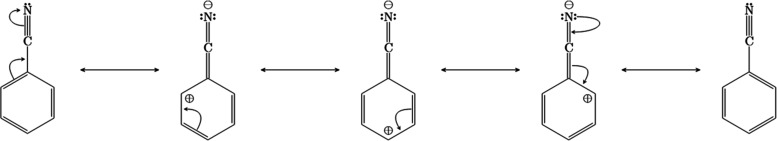
Depiction of the Mesomeric Structures of the Benzonitrile Molecule

We recall that there is one piece of data already presented in Tables 1 and 2 that was not yet analyzed. The so-called intrinsic aromatic electron sextet energy “*E* ^(*g*)^” measures the electronic energy of the pi electrons in these molecules in the field of the rest of nuclei and electrons comprising a given molecule. It is an invariant quantity within the group function approach,^73^ being not only more convenient but also more physically sound than any set of pi molecular orbital energies for these molecules. This quantity allows a comparison of the intrinsic stabilization or destabilization effects of the substituents in relation to the benzene molecule as a reference. It is insightful to compare the ordering of the calculated aromatic electron sextet energy with other properties, for which this energy should be at least partially related. First, there is the ionization potential of these molecules, which measures the energetic cost of removing an electron, counteracted by the following electronic and nuclear relaxation of the molecule.^74^ Second, there is the observed ordering of activation of the benzene ring for electrophilic aromatic substitution in the function of the substituent.^48^ A comparison between these properties is presented in Scheme 4.

**Scheme 4:**
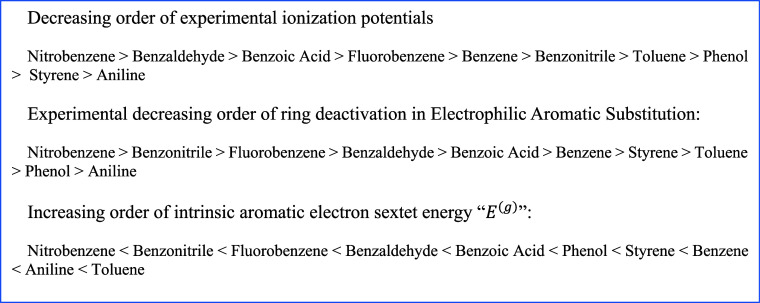
Comparison of the Ordering of Three Very Different but Somewhat Physically-Chemically Related Properties of the Molecules Calculated in This Paper

In spite of being properties of a very diverse nature, both are interpreted, at least partially, with considerations regarding the relative stability of the aromatic system as a function of the substituent. A comparison with the actual order of stability of the aromatic system, as calculated in the present paper, should contribute to clarify the relative importance of physical and chemical effects on these properties. In the following, the possible relationship between these properties and the intrinsic aromatic electron sextet energy is briefly discussed.

Regarding ionization potentials, there is only a loose correlation between its order and the order of the intrinsic aromatic electron sextet energy. This points out the importance of vibrational and electronic relaxation processes that happen upon ionization. A more detailed discussion about the ionized forms of the molecules of this work will be presented in a forthcoming paper.

Regarding the empirical scale of deactivated and activated benzene rings in electrophilic substitution reactions, there is a general better agreement between it and the intrinsic aromatic electron sextet energy scale. Particularly in the “deactivated” side of the scale, there is a perfect agreement, indicating that the intrinsic aromatic electron sextet energy is likely to encapsulate in itself the main physical effects responsible for the difficulty of an electrophile to disrupt the aromatic electron sextet. Although the intrinsic aromatic electron sextet interactions certainly play an important role for the molecules in the “activated” side of the scale, in these cases, other factors concerning the associated transition states should come into play. It is likely that in these cases, the electrophile promotes, depending on the molecule, different stabilization effects upon addition onto the aromatic ring. There are two studies on the addition of simple cations to the benzene ring using Spin-Coupled and Multi-Configuration Spin-Coupled wave functions for the pi electrons.^75,76^ While these studies offered some useful insights into the chemical bonding of Wheland intermediates, they cover a small number of very simple systems. Detailed studies with similar methods employed herein comprising a large array of model systems are soon be presented. With the data gathered so far, we believe that we have reached the limit of what can be inferred for these correlations. Nevertheless, we consider that the present discussion can provide some additional physical insights into the effects controlling both the ionization potential and the reactivity of these molecules in electrophilic substitution reactions.

The other important piece of information that can be extracted from the calculated wave functions for this set of molecules comes from the comparison between the optimized singly occupied nonorthogonal orbitals. As already pointed out above, the resulting orbitals, particularly the ones associated with sigma bonds, are visually very similar. In order to compare the effects of different substituents, the orbitals themselves may be replaced by their centroids, which is a unique point in space. The projection of these points, separately for sigma and pi orbitals, along the line connecting the ring carbon and the substituent atom directly linked to the ring hopefully should be able to inform on the releasing or withdrawing effects of the substituent. Specifically, the centroids of the optimized singly occupied nonorthogonal orbitals involving the C-X bond, where X = C, O, N, F is the atom attached to the benzene ring, are evaluated according to the scheme illustrated in Figure 9. The coordinates of each centroid are determined and projected along the direction of the C–X bond. For this projection, the origin of the coordinate system is placed at the carbon atom of the benzene ring. All projections are normalized with respect to the corresponding C–X bond length.

**Figure 9:**
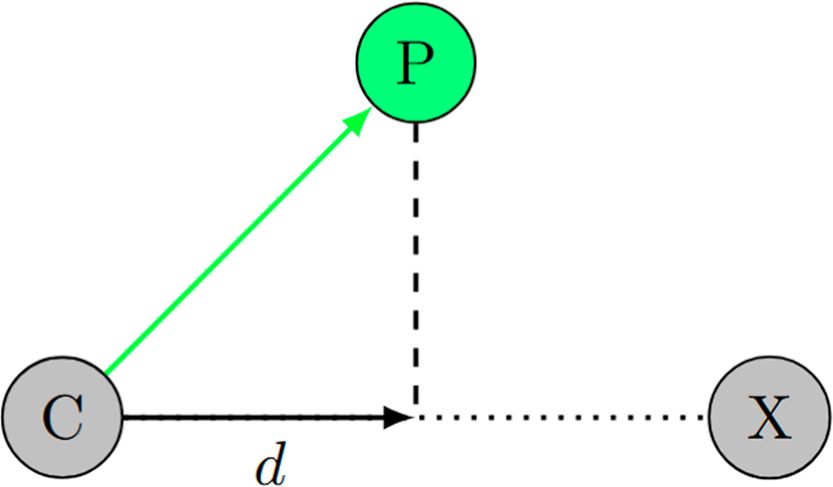
Projection scheme of the centroid coordinates (represented by the green circle P) onto the C–X bond (represented by the vector length *d*). All projections are normalized by the C–X bond length.

Figure 10 presents the projection of the centroids of the nonorthogonal singly occupied sigma and pi orbitals onto the C–X bond. Orange circles represent orbitals centered on the benzene carbon, while blue circles indicate orbitals centered on the attached X (C, O, N, and F).

**Figure 10:**
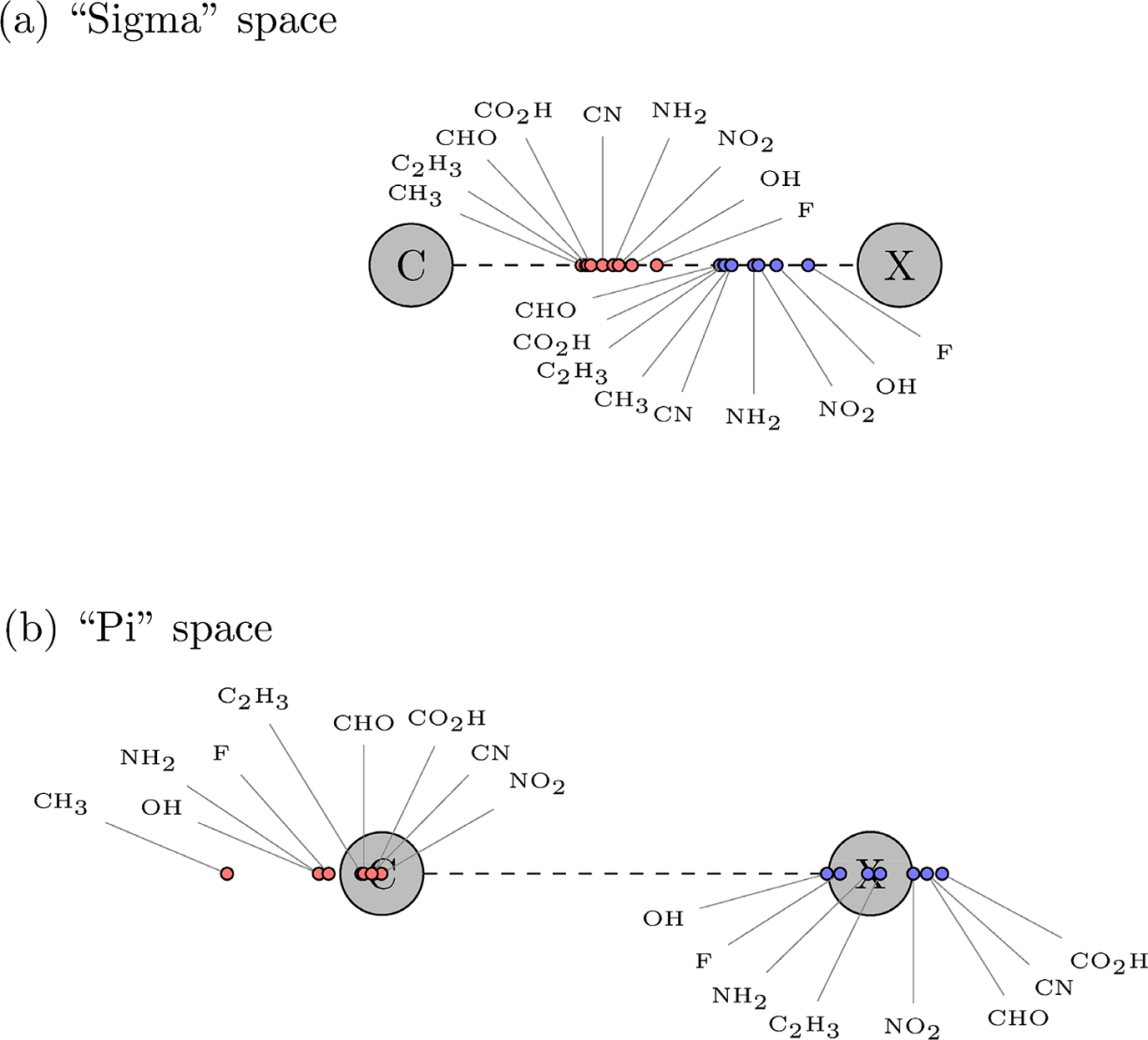
Projection of the centroids onto the C–X bond. Orange circles indicate the centroids of the orbitals centered at the benzene’s carbon, while the blue circles represent the centroids of the orbitals centered on the attached atom X. (a) Projected centroids of sigma orbitals. (b) Projected centroids of the pi orbitals. When the attached group X has more than one “pi” orbital centered in the atom adjacent to the ring, the centroid shown is for the more diffuse.

In Figure 10a, the sigma centroids most strongly displaced toward the substituent group are associated with electronegative atoms, as expected, such as F, OH, NO_2_, and NH_2_. The largest displacement is observed for fluorobenzene, followed by that of phenol and nitrobenzene. Notably, all σ orbital centroids lie within the span of the C-X bond, indicating shared electron density along the bond axis. This characteristic does not hold for the pi singly occupied nonorthogonal orbital centroids, as shown in Figure 10b. The pi centroids that lie to the far left of the benzene carbon suggest the effect of electron density being pushed toward the aromatic ring. Toluene shows the leftmost centroid. These results agree with the calculated value of “*E* ^(*g*)^” for toluene being the highest for all considered molecules, indicating it has the least stable aromatic electron sextet, likely due to the pushing electron effect of the methyl group, as shown in Figure 10b. Conversely, nitrobenzene exhibits the rightmost centroid, indicating electron density shifted toward the substituent. This is expected, being in line with the data presented in Scheme 4. Benzonitrile exhibits the second rightmost centroid. Note that benzonitrile has also the second most stable aromatic electron sextet, as can be verified in Table 2. In the last section, when discussing the calculation results for benzonitrile, it was shown that the strong orthogonal restriction in the pi space has the largest energetic penalty for this molecule, indicating that the largest stabilizing pi interaction between a substituent and the benzene ring happens for benzonitrile. Thus, the ordering in Figure 10b reflects well the electron-withdrawing strength of the substituent in the pi space, with −NO_2_ appearing as the strongest among the groups studied, followed closely by −CN and −CHO. It should be noted that the disposition of the substituent’s centroids for the pi electrons over the C and the X atoms in Figure 10b reproduces exactly the division between ortho-/para- and meta-directing groups in electrophilic aromatic substitution mechanism models. Focusing on the centroids located near the attached atom X, we find that their displacement to the right increases in the order OH < *F* < < NH_2_ < C_2_H_3_ < NO_2_ < CHO < CN < COOH. All X-centered centroids are shifted to the right of the bond. In light of the results presented in Tables 1 and 2 and the associated discussion, this fact reinforces the conclusion about the absence of actual covalent pi bonds between the substituent and the pi aromatic system of benzene.

Finally, now one can integrate all the results and analysis above and wrap up an ab initio modern valence bond-based account for the effects of the substituents on the benzene ring. Figure 11 summarizes the centroid analysis results, showing the correlation between the sigma and pi carbon-centered centroids for each compound.

**Figure 11:**
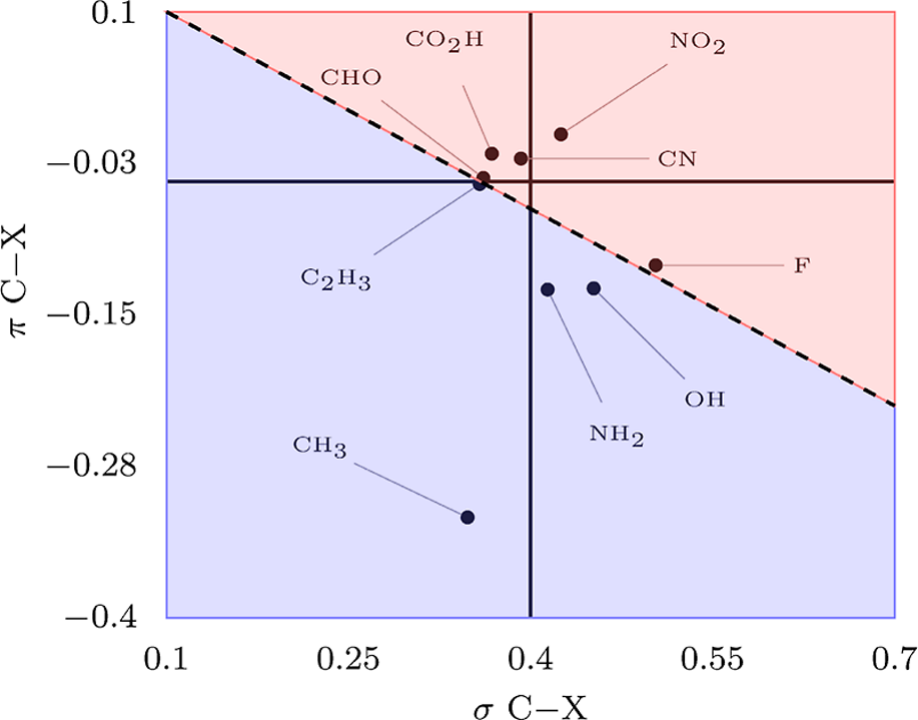
Carbon-centered centroid position of the π orbital relative to the σ orbital. The blue region spans experimentally activating groups, while deactivating groups are in the red area. The groups below the thick horizontal line have ortho-/para-orientation (pi releasing) and the groups above have the meta-orientation (pi withdrawing). The thick vertical line split the sigma releasing (left) and withdrawing (right).

First, there is the general effect of the substituent on the stability of the aromatic sextet. The most stable aromatic system is that of nitrobenzene. The nitro group is pictured from the centroid analysis above as strongly electron withdrawing in both the sigma and pi spaces. Both effects concur with the decrease of the electronic energy of the aromatic system. On the other extreme, the least stable aromatic system is that of toluene. In Figure 10a,b, one can check that in both sigma and pi spaces the centroids of the optimized singly occupied nonorthogonal sigma and pi carbon orbitals are the ones located at the most far left position, thus increasing the electron density and electronic energy of the aromatic system. In other words, due to the intrinsic strong-orthogonality of the methyl C–H bond with the aromatic electron sextet, the substituent effectively pushes the pi electrons of the ring, inducing the calculated destabilization. An interesting intermediate case is that of fluorobenzene. In the sigma space, as can be seen in Figure 10a, fluorine is the most intense electron-withdrawing substituent, contributing to indirectly stabilizing the aromatic electron sextet. On the other hand, in the pi space, as can be seen in Figure 10b, fluorine acts as an electron-releasing substituent, partly counteracting the effect on the sigma bonds. Another interesting intermediate case is that of benzoic acid. In spite of the two oxygen atoms adjacent to the atom directly bonded to the ring, such as it is the case for nitrobenzene, the stabilization of the aromatic electron sextet is less significant than the one observed in nitrobenzene. However, as can be seen in Figure 10a, the effect of the carboxyl group in the sigma space is not to withdraw electrons, just the opposite. The resultant effect of the sigma and pi spaces is of a weaker deactivating group than nitrobenzene but still directing electrophilic substitutions to the meta-positions. The position of the vinyl group, associated with the styrene molecule, in the correlation diagram of Figure 11, deserves some comments. It is distant from the other ortho/para directing groups, placed very close to the formyl group of benzaldehyde; this is in line with the fact that, from this set of molecules, styrene is the weakest activated molecule. As noted in the previous section, there is no definite agreement on the equilibrium geometry of this molecule, our calculations finding two close minima. While the results presented belong to the global minimum, it should be remarked that the usage of the secondary minimum, which is even more distant from planarity, would place the vinyl group closer to the other ortho-para-directing groups in Figure 11. All these facts concerning the effects of these substituents are in line with the empirical data present in organic chemistry textbooks. Table 3 below puts together the results obtained in this paper with the experimental data regarding the behavior of these molecules, serving as an alternative presentation of the data as presented in Figure 11.

**Table 3:** A Summary of the Calculated Sigma and Pi Effects with Data on the Reactivity in Electrophilic Aromatic Substitution Reactions^a^

	sigma	pi	experimental observations	
nitrobenzene	withdrawing	withdrawing	deactivating	meta-
benzonitrile	releasing	withdrawing	deactivating	meta-
fluorobenzene	withdrawing	releasing	deactivating	ortho-/para-
benzaldehyde	releasing	withdrawing	deactivating	meta-
benzoic acid	releasing	withdrawing	deactivating	meta-
phenol	withdrawing	releasing	activating	ortho-/para-
styrene	releasing	releasing	activating	ortho-/para-
aniline	withdrawing	releasing	activating	ortho-/para-
toluene	releasing	releasing	activating	ortho-/para-

a The molecules are ordered from the most intrinsically stable aromatic electron sextet to the least stable.

## 6 Conclusions

Full valence modern ab initio valence bond-like calculations were performed for nine monosubstituted benzene molecules. Through a progressive restriction on the spin eigenfunctions in the pi space, it was proven that in not one of these molecules there is a covalent pi bonding between substituent electrons and the aromatic electron sextet. Through the rigorous definition, within the group function approach, of an intrinsic aromatic electron sextet energy, the resulting substituent effects of energetic (des)­stabilization of the aromatic system were unambiguously quantified for the first time. Through an analysis of the centroids of the optimized singly occupied nonorthogonal sigma and pi orbitals between the substituent and the adjacent carbon atom in the benzene ring, the withdrawing/releasing effects of the substituents were comparatively evaluated, separating the effects in sigma and in pi spaces. The results presented herein shed light on the physical origin of important features of these molecules. The close relationship between the intrinsic aromatic electron sextet stabilization energy and the observed order of activation of the aromatic ring for electrophilic aromatic substitution reactions is highlighted. The degree of aromatic stabilization is precisely correlated to the observed order of deactivated rings. For activated rings, the rough correlation found seems to indicate that in these cases other factors, probably concerning the associated transition states, such as additional stabilization effects upon the electrophile addition are also relevant. The partition of the withdrawing and releasing effects of the substituents is done in a nonempirical way, stemming directly from the position of the centroids of the optimized singly occupied nonorthogonal orbitals involved in the substituent-ring bond, separated in sigma and pi spaces. Ortho-/para-directing effects are solely associated with electrons pushing in the pi space, regardless of the effects in the sigma space. Thus, a clearer rationale behind both the assignments of activation/deactivation and ortho-para/meta directing groups was obtained without resorting to Lewis resonance structures that disrupt the aromatic electron sextet. In conclusion, the results obtained gave valuable insights into the electronic structure, bonding, and properties of substituted aromatic molecules.

## Supplementary Material



## Notes

Singly occupied orbital overlap matrices, space-spin configurations overlap matrices and spin function coefficients in the Rumer basis for the pi space of all the molecules in their physically soundest wave functions at the optimized geometries; explicit expressions for the energy of group function wave functions (PDF)
