# Supplementary material for: Chemical Bonding in Monosubstituted Aromatic Molecules from Full-Valence Modern Ab Initio Valence Bond Calculations
Source: J Phys Chem A. 2025 Nov 14;129(47):11085–99. doi: 10.1021/acs.jpca.5c06234 (PMC12670402; doi:10.1021/acs.jpca.5c06234)
Supplement: Supplementary file 1 [file jp5c06234_si_001.pdf]

Supporting Information file for the paper

Chemical bonding in monosubstituted aromatic molecules from full-valence modern ab initio valence bond calculations

André G. H. Barbosa and João G. S. Monteiro*

Instituto de Química, Universidade Federal Fluminense, Niterói-RJ, Brazil

*andreghb@id.uff.br

S1. INTRODUCTION

In this Supporting Information file (section S2. WAVE FUNCTIONS FOR THE PI ELECTRONS), excerpts from the outputs from the valence bond calculations for benzoic acid, aniline, benzonitrile, benzaldehyde, styrene, phenol, fluorobenzene, nitrobenzene and toluene are shown. Additionally, (section S3.ENERGY EXPRESSIONS FOR THE GROUP ENERGY) the energy expression within the group function approach together with its relationship with an individual "group energy" is shown.

S2. WAVE FUNCTIONS FOR THE PI ELECTRONS

The cartesian coordinates for the optimized geometry for each molecule, determined at the M06-2X/6-311(d,p)++ level, are shown, together with the associated total energy. The modern Valence Bond wave function data, referring to the optimized geometries, are associated with the group of pi orbitals in the soundest wave function, indicated by

the appropriate partition (number of GVB-PP pairs, number of Spin-Coupled electrons/orbitals, number of Spin Eigenfunctions), according to the lengthy discussion presented on the paper.

For each molecule, atom labels will indicate the location of the optimized singly-occupied nonorthogonal orbitals in correspondence to the index number of the orbital overlap matrix. In all cases shown in this Supporting Information file there are five Spin Eigenfunctions (space-spin configurations named as “Normalized structure coefficients” in the VB2000 output), for which the coefficients are presented always in the same ordering corresponding to the pattern below.

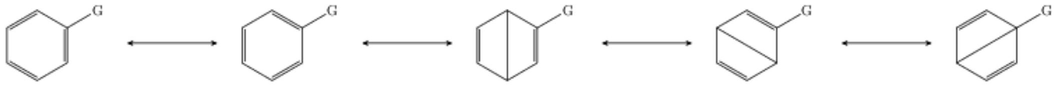

Scheme 1S. Ordered spin coupling pattern followed in the molecules herein calculated. “G” stands for the position of the substituent in relation to the spin couplings.

For the eventual pi electrons in the substituent, for the partitions presented below, they are always described with the perfect-pairing spin coupling but without orthogonality restrictions in relation to all the other singly-occupied pi orbitals.

BENZOIC ACID
Partition (18,10,5)

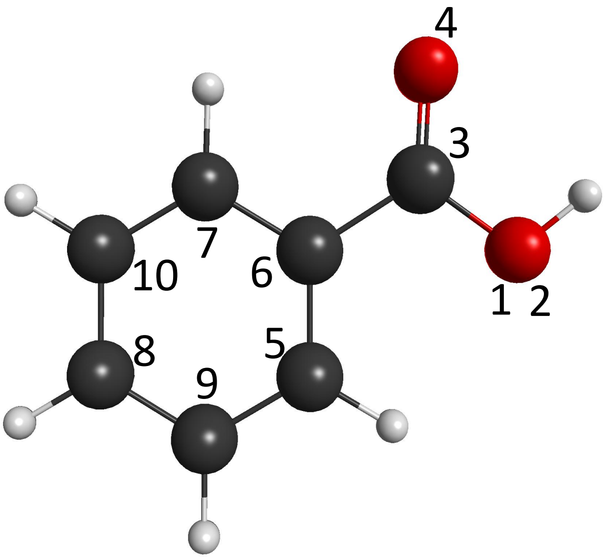

Cartesian Coordinates (Å)<br>E = -420.773479 hartree			
C	-0.030109	-2.607573	0.000000
C	0.084957	0.165116	0.000000
C	1.204636	-1.964984	0.000000
C	-1.208520	-1.864853	0.000000
C	1.266193	-0.576495	0.000000
C	-1.152338	-0.477798	0.000000
H	-0.074460	-3.690575	0.000000
C	0.094396	1.652900	0.000000
H	2.119117	-2.545176	0.000000
H	-2.167653	-2.367839	0.000000
H	2.219770	-0.064647	0.000000
H	-2.052868	0.123968	0.000000
O	-0.887817	2.345501	0.000000
O	1.339038	2.169922	0.000000
H	1.245658	3.131632	0.000000

OVERLAP MATRIX OF VB ORBITALS FOR GROUP 20

	1	2	3	4	5	6	7	8	9	10
1	1.0000									
2	0.8494	1.0000								
3	0.3651	0.1844	1.0000							
4	0.0718	0.0754	0.6984	1.0000						
5	0.0711	0.0285	0.0699	-0.0042	1.0000					
6	0.1679	0.0674	0.3072	0.0532	0.5663	1.0000				
7	0.0286	0.0133	0.0887	0.0023	0.0971	0.5094	1.0000			
8	-0.0294	-0.0121	-0.0469	-0.0014	0.0489	-0.1515	0.0363	1.0000		
9	-0.0154	-0.0056	-0.0367	-0.0068	0.5011	0.0347	-0.1444	0.5484	1.0000	
10	-0.0158	-0.0063	-0.0107	0.0008	-0.1374	0.0338	0.5560	0.4849	-0.0058	1.0000

OVERLAP MATRIX OF VB STRUCTURES FOR GROUP 20

	1	2	3	4	5
1	1.000000				
2	0.674780	1.000000			
3	-0.836758	-0.826018	1.000000		
4	-0.837771	-0.824793	0.759064	1.000000	
5	-0.838135	-0.825979	0.758732	0.758746	1.000000

HAMILTONIAN MATRIX OF VB STRUCTURES FOR GROUP 20

	1	2	3	4	5
1	-14.890708				
2	-10.096999	-14.878657			
3	12.485023	12.320222	-14.857022		
4	12.499133	12.302798	-11.344938	-14.856591	
5	12.505641	12.320802	-11.340671	-11.340820	-14.859592

Normalization constants of VB structures - i.e. N in C(norm) = N C(unnorm)
===
2.012241 1.797449 1.637394 1.633726 1.648614

====Normalized structure coefficients====
0.4902 0.3945 -0.0673 -0.0649 -0.0761

====Mulliken Weight (Chirgwin-Coulson)====
0.4563 0.3540 0.0612 0.0590 0.0695

====Inverse Overlap Weight (Gallup-Norbeck)====
0.6560 0.3097 0.0107 0.0099 0.0138

====Lowdin Weight====
0.3882 0.2063 0.1337 0.1322 0.1395

====Hiberty Weight====
0.5854 0.3792 0.0110 0.0103 0.0141

ENERGY AND DIFF OF MACROITER 55 = -418.7674801620 -0.0000004491

ANILINE
Partition (14,8,5)

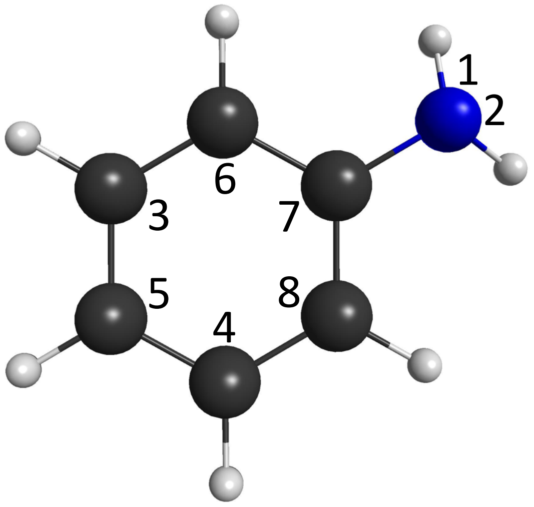

Cartesian Coordinates (Å)

E = -287.553623 hartree

C	-1.458181	-1.203161	0.071928
C	-0.070967	-1.198936	0.018215
C	-2.169346	0.001339	0.073224
C	0.632350	-0.000331	-0.030006
C	-1.458013	1.205171	0.037014
C	-0.070863	1.199274	-0.016729
N	-3.563317	0.002963	0.166782
H	1.711522	-0.001058	-0.077201
H	-2.001658	-2.140707	0.116124
H	0.467271	-2.139610	0.012316
H	-2.001597	2.143488	0.054487
H	0.467426	2.139352	-0.049917
H	-4.012439	-0.838715	-0.161720
H	-4.013580	0.830107	-0.195669

OVERLAP MATRIX OF VB ORBITALS FOR GROUP 16

	1	2	3	4	5	6	7	8
1	1.0000							
2	0.8484	1.0000						
3	-0.0376	-0.0131	1.0000					
4	-0.0378	-0.0132	0.1515	1.0000				
5	-0.0363	-0.0279	0.5522	0.5528	1.0000			
6	-0.0837	-0.0700	-0.5332	0.1262	-0.0332	1.0000		
7	0.3378	0.1993	0.0596	0.0603	-0.1610	-0.5171	1.0000	
8	0.0836	0.0701	-0.1261	0.5332	0.0341	0.0288	0.5179	1.0000

OVERLAP MATRIX OF VB STRUCTURES FOR GROUP 16

	1	2	3	4	5
1	1.000000				
2	0.681161	1.000000			
3	-0.833157	-0.832785	1.000000		
4	-0.832910	-0.833097	0.760148	1.000000	
5	-0.837848	-0.837743	0.763614	0.763674	1.000000

HAMILTONIAN MATRIX OF VB STRUCTURES FOR GROUP 16

	1	2	3	4	5
1	-10.372174				
2	-7.118344	-10.372111			
3	8.669304	8.664858	-10.342471		
4	8.666168	8.668657	-7.929898	-10.342489	
5	8.718668	8.717572	-7.967827	-7.968434	-10.347908

Normalization constants of VB structures - i.e. N in C(norm) = N C(unnorm)
==
1.590474 1.588999 1.355508 1.355496 1.390489

====Normalized structure coefficients====
0.4487 0.4483 -0.0599 -0.0599 -0.0750

====Mulliken Weight (Chirgwin-Coulson)====
0.4114 0.4108 0.0545 0.0545 0.0688

====Inverse Overlap Weight (Gallup-Norbeck)====
0.4856 0.4833 0.0086 0.0086 0.0139

====Lowdin Weight====
0.2944 0.2931 0.1315 0.1315 0.1493

====Hiberty Weight====
0.4851 0.4841 0.0086 0.0086 0.0135

ENERGY AND DIFF OF MACROITER 13 = -286.0710850984 -0.0000007374

BENZONITRILE
Partition (15,8,5)

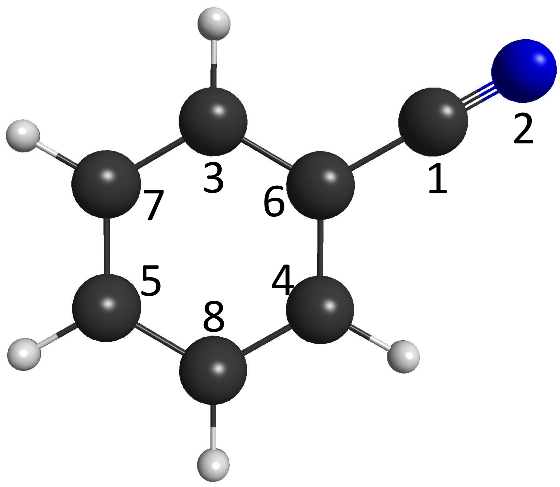

Cartesian Coordinates (Å)<br>E = -324.437732 hartree			
C	1.538370	-1.202980	0.000000
C	2.231210	0.003320	0.000000
C	0.150990	-1.217500	0.000000
C	1.538620	1.209620	0.000000
C	-0.541870	-0.005290	0.000000
C	0.151170	1.207170	0.000000
H	-0.403050	2.134350	0.000000
H	3.313250	-0.000750	0.000000
H	-0.396150	-2.152640	0.000000
H	2.075450	2.151130	0.000000
C	-1.979160	-0.000920	0.000000
H	2.082580	-2.136400	0.000000
N	-3.129310	0.005950	0.000000

OVERLAP MATRIX OF VB ORBITALS FOR GROUP 17

	1	2	3	4	5	6	7	8
1	1.0000							
2	0.6854	1.0000						
3	0.0925	-0.0153	1.0000					
4	0.0935	-0.0154	0.0801	1.0000				
5	0.0515	-0.0055	-0.0043	-0.0070	1.0000			
6	-0.3789	-0.0796	-0.5353	-0.5379	-0.1488	1.0000		
7	0.0312	0.0072	-0.5159	0.1880	0.4999	0.0235	1.0000	
8	-0.0295	-0.0070	-0.1800	0.5142	-0.5080	-0.0294	0.0580	1.0000

OVERLAP MATRIX OF VB STRUCTURES FOR GROUP 17

	1	2	3	4	5
1	1.000000				
2	0.673338	1.000000			
3	-0.828433	-0.830990	1.000000		
4	-0.831806	-0.827752	0.754235	1.000000	
5	-0.830187	-0.829125	0.754758	0.754147	1.000000

HAMILTONIAN MATRIX OF VB STRUCTURES FOR GROUP 17

	1	2	3	4	5
1	-10.049229				
2	-6.819418	-10.048474			
3	8.353584	8.377304	-10.020543		
4	8.385872	8.346616	-7.626970	-10.020896	
5	8.370256	8.359360	-7.631123	-7.625360	-10.021025

Normalization constants of VB structures - i.e. N in C(norm) = N C(unnorm)
==
1.501838 1.489812 1.296759 1.305733 1.267825

====Normalized structure coefficients====
0.4414 0.4355 -0.0724 -0.0742 -0.0712

====Mulliken Weight (Chirgwin-Coulson)====
0.4041 0.3978 0.0659 0.0675 0.0648

====Inverse Overlap Weight (Gallup-Norbeck)====
0.4918 0.4681 0.0136 0.0146 0.0120

====Lowdin Weight====
0.2984 0.2859 0.1406 0.1443 0.1307

====Hiberty Weight====
0.4867 0.4738 0.0131 0.0137 0.0127

ENERGY AND DIFF OF MACROITER 25 = -322.7997328723 -0.0000008781

BENZALDEHYDE
Partition (16,8,5)

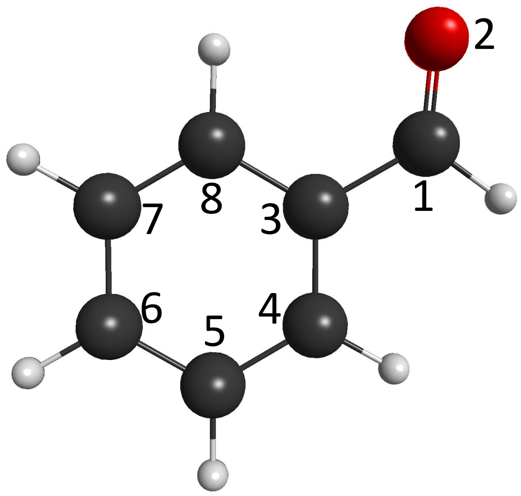

Cartesian Coordinates (Å) E = -345.516000 hartree			
C	0.484180	0.226760	0.000000
C	0.023680	-1.090510	0.000000
C	-0.417000	1.289430	0.000000
C	-1.340450	-1.332920	0.000000
C	-1.785620	1.045330	0.000000
C	-2.241010	-0.268060	0.000000
C	1.940560	0.502920	0.000000
O	2.793620	-0.345310	0.000000
H	0.748640	-1.897480	0.000000
H	-0.042440	2.306440	0.000000
H	-1.711910	-2.348490	0.000000
H	-2.491690	1.868240	0.000000
H	-3.303820	-0.468770	0.000000
H	2.210470	1.575530	0.000000

OVERLAP MATRIX OF VB ORBITALS FOR GROUP 18

	1	2	3	4	5	6	7	8
1	1.0000							
2	0.6582	1.0000						
3	0.3497	0.0569	1.0000					
4	0.0955	-0.0081	0.6178	1.0000				
5	0.0624	0.0122	-0.0210	-0.4226	1.0000			
6	-0.0548	-0.0006	-0.1489	0.0023	-0.5799	1.0000		
7	-0.0211	-0.0062	-0.0885	0.1080	-0.0555	-0.3954	1.0000	
8	-0.0928	-0.0001	-0.4777	-0.1362	-0.1464	-0.0052	0.6099	1.0000

OVERLAP MATRIX OF VB STRUCTURES FOR GROUP 18

	1	2	3	4	5
1	1.000000				
2	0.665009	1.000000			
3	-0.840274	-0.809955	1.000000		
4	-0.846034	-0.805887	0.753216	1.000000	
5	-0.844103	-0.809515	0.749928	0.753141	1.000000

HAMILTONIAN MATRIX OF VB STRUCTURES FOR GROUP 18

	1	2	3	4	5
1	-10.135556				
2	-6.787227	-10.098206			
3	8.540067	8.219190	-10.091333		
4	8.596053	8.180008	-7.672748	-10.090720	
5	8.578408	8.216485	-7.640969	-7.672993	-10.094691

Normalization constants of VB structures - i.e. N in C(norm) = N C(unnorm)
==
1.704320 1.233643 1.258043 1.256970 1.269077

====Normalized structure coefficients====
-0.5441 -0.2890 0.0867 0.0768 0.0936

====Mulliken Weight (Chirgwin-Coulson)====
0.5186 0.2481 0.0786 0.0696 0.0852

====Inverse Overlap Weight (Gallup-Norbeck)====
0.8595 0.0978 0.0145 0.0113 0.0169

====Lowdin Weight====
0.5492 0.0937 0.1176 0.1159 0.1237

====Hiberty Weight====
0.7369 0.2079 0.0187 0.0147 0.0218

ENERGY AND DIFF OF MACROITER 6 = -343.8188262693 -0.0000003246

STYRENE
Partition (16,8,5)

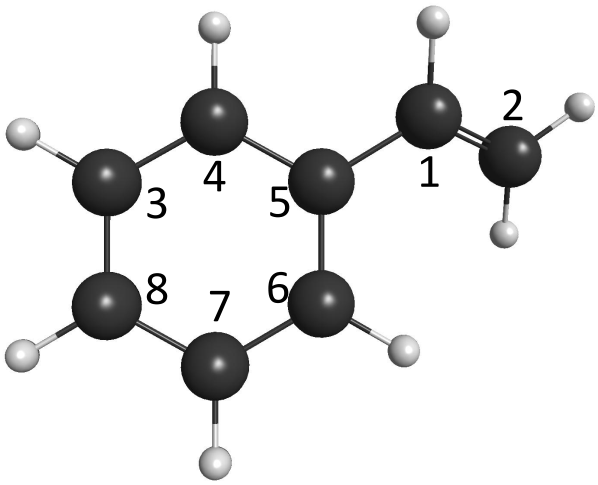

Cartesian Coordinates (Å)

E = -309.603357 hartree

C	-0.356911	-1.176789	0.024744
C	0.350645	1.111888	0.083371
C	0.951038	-1.583839	0.221431
C	1.663014	0.706841	0.281339
C	1.967739	-0.643784	0.352253
C	-2.055567	0.664446	-0.243254
C	-3.162159	-0.069293	-0.266397
H	-1.133478	-1.921641	-0.084401
H	0.115277	2.167619	0.027926
H	1.182360	-2.639407	0.269707
H	2.445998	1.446600	0.378821
H	2.988927	-0.965230	0.504614
H	-2.144859	1.738043	-0.375346
H	-3.151356	-1.142370	-0.129180
H	-4.127472	0.391082	-0.420610

OVERLAP MATRIX OF VB ORBITALS FOR GROUP 18

	1	2	3	4	5	6	7	8
1	1.0000							
2	0.6412	1.0000						
3	-0.0488	-0.0171	1.0000					
4	0.0980	-0.0157	0.4817	1.0000				
5	0.4015	0.0975	0.0302	0.5655	1.0000			
6	-0.0934	0.0031	0.1434	-0.0247	-0.4550	1.0000		
7	0.0044	0.0027	-0.0472	0.1452	-0.0140	0.5730	1.0000	
8	0.0480	-0.0034	-0.5801	-0.0405	0.1621	0.0196	0.4735	1.0000

OVERLAP MATRIX OF VB STRUCTURES FOR GROUP 18

	1	2	3	4	5
1	1.000000				
2	0.674102	1.000000			
3	-0.822258	-0.841402	1.000000		
4	-0.820145	-0.842084	0.760535	1.000000	
5	-0.820610	-0.842519	0.759980	0.758494	1.000000

HAMILTONIAN MATRIX OF VB STRUCTURES FOR GROUP 18

	1	2	3	4	5
1	-9.580916				
2	-6.520989	-9.603912			
3	7.912046	8.103326	-9.564615		
4	7.891714	8.110568	-7.343178	-9.565469	
5	7.897934	8.115384	-7.339002	-7.325037	-9.569094

Normalization constants of VB structures - i.e. N in C(norm) = N C(unnorm)
==
1.312295 1.599992 1.243773 1.246982 1.266881

====Normalized structure coefficients====
0.3466 0.5218 -0.0654 -0.0707 -0.0864

====Mulliken Weight (Chirgwin-Coulson)====
0.3054 0.4920 0.0595 0.0643 0.0789

====Inverse Overlap Weight (Gallup-Norbeck)====
0.1945 0.7687 0.0091 0.0108 0.0169

====Lowdin Weight====
0.1498 0.4612 0.1245 0.1268 0.1378

====Hiberty Weight====
0.2936 0.6655 0.0105 0.0122 0.0182

ENERGY AND DIFF OF MACROITER 25 = -307.9553016739 -0.0000006315

PHENOL
Partition (14,8,5)

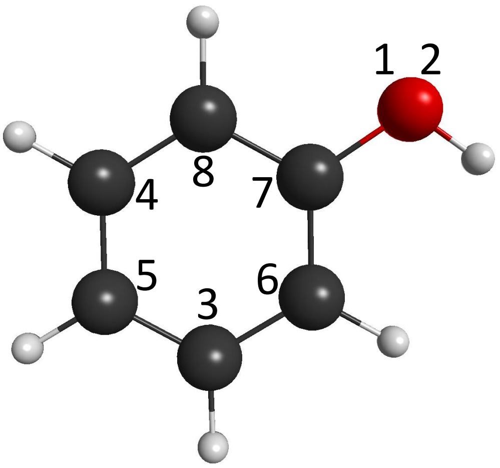

Cartesian Coordinates (Å)<br>E = -307.423088 hartree			
C	-1.159220	-1.200750	0.000000
C	-1.874170	-0.005590	0.000000
C	0.227430	-1.202560	0.000000
C	-1.177730	1.196410	0.000000
C	0.916940	0.006510	0.000000
C	0.213550	1.207640	0.000000
H	0.750410	2.148980	0.000000
H	-2.957200	-0.016210	0.000000
H	0.792590	-2.125300	0.000000
H	-1.712630	2.137860	0.000000
O	2.278280	-0.053730	0.000000
H	-1.685250	-2.145550	0.000000
H	2.648140	0.831550	0.000000

OVERLAP MATRIX OF VB ORBITALS FOR GROUP 16

```
      1        2        3        4        5        6        7        8
 1 1.0000.
 2 0.8604 1.0000
 3-0.0445-0.0121 1.0000
 4-0.0277-0.0070 0.1281 1.0000
 5-0.0442-0.0262 0.5820 0.5094 1.0000
 6 0.0852 0.0694 0.5173-0.1329 0.0636 1.0000
 7 0.3589 0.1974 0.0983 0.0276-0.1435 0.5646 1.0000
 8 0.0965 0.0683-0.1315 0.5454-0.0003-0.0190 0.4730 1.0000
```

OVERLAP MATRIX OF VB STRUCTURES FOR GROUP 16

	1	2	3	4	5
1	1.000000				
2	0.677436	1.000000			
3	-0.836366	-0.823477	1.000000		
4	-0.839533	-0.827271	0.757813	1.000000	
5	-0.842629	-0.827213	0.758259	0.764777	1.000000

HAMILTONIAN MATRIX OF VB STRUCTURES FOR GROUP 16

	1	2	3	4	5
1	-10.632217				
2	-7.251674	-10.616172			
3	8.917415	8.774553	-10.595810		
4	8.950429	8.814338	-8.098841	-10.595958	
5	8.983598	8.814809	-8.104780	-8.173058	-10.598480

Normalization constants of VB structures - i.e. N in C(norm) = N C(unnorm)
==
1.693883 1.489688 1.364845 1.361770 1.379038

====Normalized structure coefficients====
-0.5069 -0.3817 0.0701 0.0618 0.0709

====Mulliken Weight (Chirgwin-Coulson)====
0.4744 0.3408 0.0637 0.0563 0.0648

====Inverse Overlap Weight (Gallup-Norbeck)====
0.6940 0.2746 0.0114 0.0085 0.0115

====Lowdin Weight====
0.4077 0.1919 0.1319 0.1299 0.1386

====Hiberty Weight====
0.6170 0.3499 0.0118 0.0092 0.0121

ENERGY AND DIFF OF MACROITER 40 = -305.9041139802 -0.0000009801

FLUOROBENZENE
Partition (14,8,5)

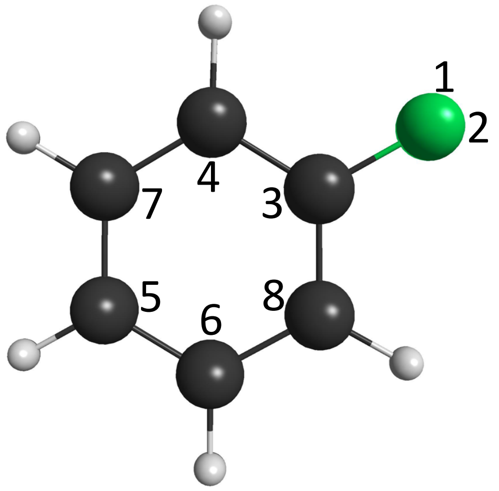

Cartesian Coordinates (Å)<br>E = -331.440685 hartree			
C	-1.196140	1.203010	0.000000
C	-1.898360	0.002520	0.000000
C	0.194120	1.214980	0.000000
C	-1.205240	-1.203410	0.000000
C	0.855400	0.000460	0.000000
C	0.185110	-1.209640	0.000000
H	0.752150	-2.128760	0.000000
H	-2.980230	0.011390	0.000000
H	0.760400	2.137760	0.000000
H	-1.742170	-2.144860	0.000000
F	2.200230	-0.005860	0.000000
H	-1.733120	2.140800	0.000000

OVERLAP MATRIX OF VB ORBITALS FOR GROUP 16

```
        1        2        3        4        5        6        7        8
 1 1.0000
 2 0.8777 1.0000
 3 0.2963 0.1401 1.0000
 4 0.0861 0.0474 0.5504 1.0000
 5 0.0357 0.0313 0.1028-0.0432 1.0000
 6-0.0160-0.0146 0.1174-0.1084-0.5376 1.0000
 7-0.0163-0.0148 0.1184 0.5413-0.5386 0.0985 1.0000
 8 0.0861 0.0473 0.5495 0.0657-0.0437 0.5420-0.1068 1.0000
```

OVERLAP MATRIX OF VB STRUCTURES FOR GROUP 16

	1	2	3	4	5
1	1.000000				
2	0.678374	1.000000			
3	-0.833971	-0.834792	1.000000		
4	-0.834570	-0.834101	0.763358	1.000000	
5	-0.833575	-0.833763	0.763010	0.763014	1.000000

HAMILTONIAN MATRIX OF VB STRUCTURES FOR GROUP 16

	1	2	3	4	5
1	-11.165742				
2	-7.628074	-11.166005			
3	9.339823	9.349627	-11.139091		
4	9.346985	9.341310	-8.571004	-11.138889	
5	9.335106	9.337292	-8.566715	-8.566672	-11.136724

Normalization constants of VB structures - i.e. N in C(norm) = N C(unnorm)
==
1.622540 1.625315 1.400518 1.399170 1.390320

====Normalized structure coefficients====
-0.4428 -0.4450 0.0713 0.0703 0.0631

====Mulliken Weight (Chirgwin-Coulson)====
0.4054 0.4077 0.0652 0.0643 0.0575

====Inverse Overlap Weight (Gallup-Norbeck)====
0.4793 0.4862 0.0126 0.0122 0.0097

====Lowdin Weight====
0.2916 0.2948 0.1400 0.1393 0.1343

====Hiberty Weight====
0.4805 0.4851 0.0125 0.0121 0.0098

ENERGY AND DIFF OF MACROITER 65 = -329.8998844047 -0.0000009276

NITROBENZENE
Partition (18,10,5)

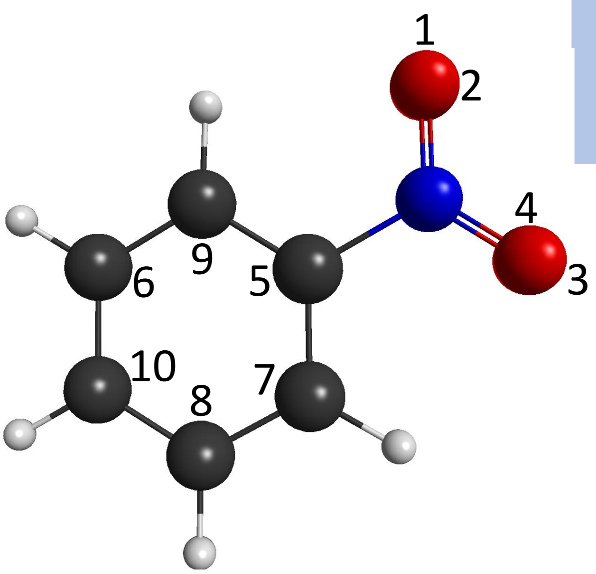

Cartesian Coordinates (Å)<br>E = -436.688171 hartree			
C	-0.000000	-2.568665	0.000000
C	-0.000000	0.174515	0.000000
C	1.209056	-1.877239	0.000000
C	-1.209056	-1.877239	0.000000
C	1.218182	-0.488078	0.000000
C	-1.218182	-0.488078	0.000000
H	-0.000000	-3.652182	0.000000
N	0.000000	1.655435	0.000000
H	2.146452	-2.418948	0.000000
H	-2.146452	-2.418948	0.000000
H	2.138140	0.080157	0.000000
H	-2.138140	0.080157	0.000000
O	-1.074985	2.214106	0.000000
O	1.074985	2.214106	0.000000

OVERLAP MATRIX OF VB ORBITALS FOR GROUP 20

```
       1        2        3        4        5        6        7        8        9        10
 1 1.0000
 2 0.7052 1.0000
 3 0.2303 0.2602 1.0000
 4 0.2600 0.6914 0.7053 1.0000
 5 0.0915 0.3084 0.0915 0.3084 1.0000
 6-0.0031-0.0243-0.0087-0.0340 0.0280 1.0000
 7 0.0166 0.0710 0.0247 0.1006 0.5470-0.1751 1.0000
 8-0.0086-0.0337-0.0030-0.0241 0.0289-0.0658 0.5170 1.0000
 9-0.0247-0.1006-0.0166-0.0710-0.5469-0.5164-0.1014 0.1751 1.0000
10 0.0135 0.0596 0.0135 0.0596 0.1811-0.4943 0.0127-0.4943-0.0134 1.0000
```

OVERLAP MATRIX OF VB STRUCTURES FOR GROUP 20

	1	2	3	4	5
1	1.000000				
2	0.673632	1.000000			
3	-0.826591	-0.832386	1.000000		
4	-0.832319	-0.826627	0.754787	1.000000	
5	-0.830135	-0.830121	0.753599	0.753554	1.000000

HAMILTONIAN MATRIX OF VB STRUCTURES FOR GROUP 20

	1	2	3	4	5
1	-15.499548				
2	-10.494816	-15.499578			
3	12.840766	12.929069	-15.470958		
4	12.928028	12.841326	-11.746430	-15.470951	
5	12.894751	12.894564	-11.728367	-11.727674	-15.471505

Normalization constants of VB structures - i.e. N in C(norm) = N C(unnorm)
===
1.500774 1.501193 1.294954 1.294897 1.293793

====Normalized structure coefficients====
-0.4387 -0.4389 0.0727 0.0727 0.0716

====Mulliken Weight (Chirgwin-Coulson)====
0.4012 0.4014 0.0661 0.0661 0.0651

====Inverse Overlap Weight (Gallup-Norbeck)====
0.4797 0.4806 0.0135 0.0135 0.0128

====Lowdin Weight====
0.2920 0.2925 0.1386 0.1386 0.1383

====Hiberty Weight====
0.4802 0.4807 0.0132 0.0132 0.0128

ENERGY AND DIFF OF MACROITER 36 = -434.6432416922 -0.0000009059

TOLUENE
Partition (15,6,5)

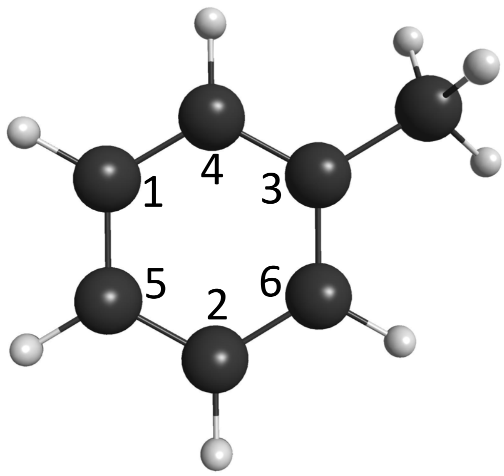

Cartesian Coordinates (Å)<br>**E = -271.505244 hartree**			
C	-1.409957	-1.196775	0.111382
C	-0.020771	-1.201144	0.058933
C	-2.124174	0.002630	0.102783
C	0.676427	0.000275	-0.003315
C	-1.411041	1.200465	0.045835
C	-0.020873	1.202336	-0.006961
H	-3.962672	-0.068272	1.214045
C	-3.629705	-0.001141	0.175649
H	1.756358	-0.001009	-0.052108
H	-1.952509	-2.135257	0.156462
H	0.521052	-2.139716	0.060852
H	-1.953017	2.140161	0.039699
H	0.520520	2.139837	-0.057528
H	-4.050228	-0.853222	-0.362241
H	-4.049873	0.911767	-0.249039

OVERLAP MATRIX OF VB ORBITALS FOR GROUP 17

	1	2	3	4	5	6
1	1.0000					
2	-0.0338	1.0000				
3	-0.0022	-0.0008	1.0000			
4	0.5245	0.1885	0.5014	1.0000		
5	0.5197	-0.5333	-0.1931	-0.0021	1.0000	
6	-0.1888	-0.5114	0.5184	-0.0317	-0.0006	1.0000

OVERLAP MATRIX OF VB STRUCTURES FOR GROUP 17

	1	2	3	4	5
1	1.000000				
2	0.677533	1.000000			
3	-0.834068	-0.829543	1.000000		
4	-0.832430	-0.831555	0.757742	1.000000	
5	-0.833871	-0.830782	0.757657	0.758129	1.000000

HAMILTONIAN MATRIX OF VB STRUCTURES FOR GROUP 17

	1	2	3	4	5
1	-6.403978				
2	-4.391464	-6.400606			
3	5.368310	5.338877	-6.374145		
4	5.358466	5.350919	-4.898799	-6.373912	
5	5.367540	5.346625	-4.898714	-4.901627	-6.374589

Normalization constants of VB structures - i.e. N in C(norm) = N C(unnorm)
==
1.307161 1.272463 1.111529 1.110020 1.114129

====Normalized structure coefficients====
-0.4468 -0.4202 0.0751 0.0739 0.0773

====Mulliken Weight (Chirgwin-Coulson)====
0.4111 0.3828 0.0684 0.0673 0.0705

====Inverse Overlap Weight (Gallup-Norbeck)====
0.5253 0.4309 0.0145 0.0140 0.0154

====Lowdin Weight====
0.3152 0.2689 0.1383 0.1374 0.1402

====Hiberty Weight====
0.5076 0.4490 0.0143 0.0139 0.0152

ENERGY AND DIFF OF MACROITER 16 = -270.0671891105 -0.0000006976

S3.ENERGY EXPRESSIONS FOR THE GROUP ENERGY

The group function approach approximates a many-electron wave function by expressing it as product of strong orthogonal sub wave functions, each one associated with a disjoint subset of the total number of electrons.[1, 2] The product is antisymmetrized and in the case of the present paper, can be expressed by Equation (1) in the main text. In our discussion a special importance was attributed to the Spin-Coupled group within a specific partition (see Tables 1 and 2), containing just the aromatic electron sextet. In these cases, one can isolate an "intrinsic aromatic electron sextet energy", named "$E^{(g)}$" for all the molecules, which allows a comparison between the different electronic effects of the different molecular environments in the aromatic system. A partition of the electronic density within the group function approach that allows a clear interpretation of the interplay between physical effects defining an optimized wave function is presented in the work of Cardozo and Nascimento.[3] In the present work we found to be expedient to express the group energies in a different but equivalent way, using explicitly the space-spin configurations defined in Equation (1) as a basis, which allows the more straightforward definition of the individual group energy showed in equations (3) and (4) in the main text.

For an expansion comprising "n_g" groups, with each group "(g)" containing "$N^{(g)}$" orbitals for "$N^{(g)}$" electrons, the total electronic energy can be compactly written in terms of the space-spin configurations as a sum of three terms:

$$E = E_{intragroup} + E_{intergroup(Coulomb)} + E_{intergroup(Exchange)} \qquad (S1)$$

The intragroup term describes the electronic energy of each group in the field of the other groups, all subjected to the same nuclear repulsion term for fixed point nuclei in the Born-Oppenheimer approximation.

$$E_{intragroup} = \sum_{g=1}^{n_g} \left\{ \frac{1}{\Delta^{(g)}} \sum_{k=1}^{n_{conf}^{(g)}} \sum_{l=1}^{n_{conf}^{(g)}} c_k^{(g)} c_l^{(g)} \left[\sum_{\mu,\nu=1}^{N^{(g)}} D_{kl}^{(g)}(\mu|\nu) \langle \psi_\mu | \hat{h}_1 | \psi_\nu \rangle^{(g)} + \frac{1}{2} \sum_{\mu,\nu,\sigma,\tau=1}^{N^{(g)}} D_{kl}^{(g)}(\mu\nu|\sigma\tau) \langle \psi_\mu \psi_\nu | \hat{g}_2 | \psi_\sigma \psi_\tau \rangle^{(g)} \right] \right\} \quad (S2)$$

The factor between square brackets is the Hamiltonian matrix element "H_{kl}" between space-spin configurations "k" and "l", compactly presented in Equation (3) of the main text. "$\hat{h}_1$" stands for the one-electron operators associated with the electron's kinetic energies and electron-nucleus attractions. "$\hat{g}_2$" stands for the electron-electron repulsion operator. "$D_{kl}^{(g)}(\mu|\nu)$" is the one-particle density matrix element between space-spin configurations "k" and "l" associated with orbitals "μ" and "ν". "$D_{kl}^{(g)}(\mu\nu|\sigma\tau)$" is the two-particle density matrix element between space-spin configurations "k" and "l" associated with orbitals "μ", "ν", "σ" and "τ". The normalization term "$\Delta^{(g)}$" is described in Equation (4) of the main text. In the case of a closed-shell group defined by doubly occupied spin orbitals there is only one space-spin configuration and the density matrix elements have fixed values.[4] In the case of GVB-PP groups, there is also only one space-spin configuration, but the density matrix elements have to be determined in each case. A GVB-PP group is a special case of a general Spin-Coupled group. Spin-Coupled groups can comprise more than one space-spin configuration; formulas for the density matrix elements for these cases can be found in the original Gerratt's paper on the theory.[5]

The additional terms of Equation (S1) are associated with intergroup contributions for the total energy. Since groups are strong-orthogonal to each other there are no intergroup one-electron contributions. Even so, it should be noted that the two-particle

density elements of the intergroup terms can always be factored in terms of one-particle density elements belonging to different groups, and so they are presented in this way.

$$E_{intergroup(Coulomb)}$$
$$= \frac{1}{2}\sum_{\substack{g,h=1\\(g\neq h)}}^{n_g}\left\{\frac{1}{2}\frac{1}{\sqrt{\Delta^{(g)}\Delta^{(h)}}}\sum_{k=1}^{n_{conf}^{(g)}}\sum_{l=1}^{n_{conf}^{(h)}} c_k^{(g)} c_l^{(h)}\left[\sum_{\mu=1}^{N^{(g)}}\sum_{\nu=1}^{N^{(h)}} D_{kk}^{(g)}(\mu|\mu)D_{ll}^{(h)}(\nu|\nu)\langle\psi_\mu\psi_\nu|\hat{g}_2|\psi_\mu\psi_\nu\rangle^{(g,h)}\right]\right\}$$

$$(S3)$$

The "$E_{intergroup(Coulomb)}$" term describes the electrostatic repulsion between electrons in different groups.

$$E_{intergroup(Exchange)}$$
$$= -\frac{1}{2}\sum_{\substack{g,h=1\\(g\neq h)}}^{n_g}\left\{\frac{1}{4}\frac{1}{\sqrt{\Delta^{(g)}\Delta^{(h)}}}\sum_{k=1}^{n_{conf}^{(g)}}\sum_{l=1}^{n_{conf}^{(h)}} c_k^{(g)} c_l^{(h)}\left[\sum_{\substack{\mu,\nu=1\\\mu\neq\nu}}^{N^{(g)}}\sum_{\substack{\sigma,\tau=1\\\sigma\neq\tau}}^{N^{(h)}} D_{kk}^{(g)}(\mu|\nu)D_{ll}^{(h)}(\sigma|\tau)\langle\psi_\mu\psi_\sigma|\hat{g}_2|\psi_\nu\psi_\tau\rangle^{(g,h)}\right]\right\}$$

$$(S4)$$

The "$E_{intergroup(Exchange)}$" term corrects the description of the electrostatic repulsion between electrons in different groups preventing their double counting due to the total wave function antisymmetrization.

REFERENCES

(1) McWeeny, R. Some Recent Advances in Density Matrix Theory. *Reviews of Modern Physics* **1960**, *32* (2), 335-369. DOI: 10.1103/RevModPhys.32.335.
(2) McWeeny, R. *Methods of Molecular Quantum Mechanics*; Academic Press, 1989.
(3) Cardozo, T. M.; Nascimento, M. A. C. Energy partitioning for generalized product functions: The interference contribution to the energy of generalized valence bond and spin coupled wave functions. *Journal of Chemical Physics* **2009**, *130* (10). DOI: 10.1063/1.3085953.
(4) Bobrowicz, F. W.; Goddard, W. A. The Self-Consistent Field Equations for Generalized Valence Bond and Open-Shell Hartree—Fock Wave Functions. in *Methods of Electronic Structure Theory*, Schaeffer, H. F. Ed.; Plenum, 1977; pp 79-127.
(5) Gerratt, J. General Theory of Spin-Coupled Wave Functions for Atoms and Molecules. In *Advances in Atomic and Molecular Physics*, Bates, D. R., Esterman, I. Eds.; Vol. 7; Academic Press, 1971; pp 141-221.
